# Political Narratives and the US Partisan Gender Gap

**DOI:** 10.3389/fpsyg.2021.675684

**Published:** 2021-06-18

**Authors:** Armenak Antinyan, Thomas Bassetti, Luca Corazzini, Filippo Pavesi

**Affiliations:** ^1^Wenlan School of Business, Zhongnan University of Economics and Law, Wuhan, China; ^2^Cardiff Business School, Cardiff University, Cardiff, United Kingdom; ^3^Department of Economics and Management “Marco Fanno”, University of Padua, Padua, Italy; ^4^Department of Economics and VERA (Venice Centre in Economic and Risk Analytics for Public Policies), University of Venice “Ca' Foscari”, Venezia, Italy; ^5^School of Economics and Management, University “Carlo Cattaneo” - LIUC, Castellanza, Italy; ^6^Stevens Institute of Technology, School of Business, Hoboken, NJ, United States

**Keywords:** political narrative, COVID-19, Partisan Gender Gap, survey experiment, polarization

## Abstract

Social scientists have devoted considerable research effort to investigate the determinants of the Partisan Gender Gap (PGG), whereby US women (men) tend to exhibit more liberal (conservative) political preferences over time. Results of a survey experiment run during the COVID-19 emergency and involving 3,086 US residents show that exposing subjects to alternative narratives on the causes of the pandemic increases the PGG: relative to a baseline treatment in which no narrative manipulation is implemented, exposing subjects to either the *Lab narrative* (claiming that COVID-19 was caused by a lab accident in Wuhan) or the *Nature narrative* (according to which COVID-19 originated in the wildlife) makes women more liberal. The polarization effect documented in our experiment is magnified by the political orientation of participants' state of residence: the largest PGG effect is between men residing in Republican-leaning states and women living in Democratic-leaning states.

**JEL Classification:** J16, D83, C83, C99, P16, D72.

## Introduction

Political polarization is a central question in the United States as it can affect the design and implementation of various (social) policies, as well as on the general functioning of democracy (Bail et al., 2018). Political polarization is mainly attributed to the partisan identification of US citizens (Bail et al., 2018; Peterson and Iyengar, 2020), as it is a much stronger predictor of the policy preferences of Americans than any other socio-demographic variable (Dimock et al., 2014).^1^

Regarding partisan identification, the *partisan gender gap* (PGG)—i.e., the tendency of females to be more Democratic than males—is an important feature of the US political landscape. To this date, the PGG has been investigated mainly through socio-economic determinants, such as gender differences in policy preferences (Shapiro and Mahajan, 1986; Kaufmann and Petrocik, 1999),^2^ socio-demographic conditions such as being single or divorced (Edlund and Pande, 2002),^3^ feminism (Conover, 1988), cultural values (Kaufmann, 2002), and economic autonomy (Huddy et al., 2008). More recently, Clark (2017) and Gillion et al. (2020) have shown that at least part of the PGG is the consequence of an ideological sorting mechanism. According to Gillion et al. (2020), men and women initially selected the party that matched their policy preferences, then this pre-existing sorting fueled the PGG over time, leading to a gap not fully explained by differences in policy opinions.

In this paper, first, we go beyond socio-economic determinants and investigate the impact of political narratives on the PGG (in the United States), which to the best of our knowledge is an understudied question in the literature. Indeed, in the social science literature there is an increasing interest in understanding how narratives form and influence opinions and behaviors (Morson and Schapiro, 2017; Shiller, 2017, 2019), and our work represents a contribution to this stream. Formally, we test the following hypothesis: because political narratives make the partisan affiliation salient (contain partisan cues), and given the existing gender gap in partisanship, we expect that political narratives will increase political polarization between men and women (i.e., the PGG widens). Second, we investigate the mechanisms through which the political narratives affect the PGG. We identify three potential mechanisms (one rational and two behavioral) through which the political narratives can affect the PGG.

As for the rational (or Bayesian) explanations, recent theoretical and experimental contributions show that individuals receiving the same informative signal on an unknown state of the world may rationally develop polarized beliefs on the same state if they start from heterogeneous priors or have differing private information (Andreoni and Mylovanov, 2012; Baliga et al., 2013; Fryer et al., 2019; Loh and Phelan, 2019; Eliaz and Spiegler, 2020). For example, Andreoni and Mylovanov (2012) consider a model in which information has a private and a public dimension, and both dimensions are important for identifying the state of nature. In this context, the heterogeneous beliefs about the state of the world determined by private information may influence the interpretation of public information and may cause ex-post polarization. Eliaz and Spiegler (2020) instead present a model of competing narratives in a Bayesian framework and represent narratives as causal relations that map actions into consequences. They provide a theoretical foundation for the emergence of false narratives that maximize anticipatory utility by providing easy solutions to complex issues. However, these narratives necessarily also require the co-existence of rational (or “correct”) versions of the facts in order to thrive, suggesting that polarization of opinions is an equilibrium feature. These models are consistent with empirical evidence showing that the exposure of contending factions to the same objective empirical evidence can lead to social positions that are politically polarized (Lord et al., 1979). Thus, even if individuals disregard political cues and adopt “accuracy-driven reasoning,” in the sense that they make use of cognitive resources to accurately evaluate information (Kunda, 1990; Gilens, 2001; Howell and West, 2009), if gender determines distinct initial views of the world (i.e., beliefs on the state of the world), narratives may rationally lead males and females to update these positions in opposite directions.

As for behavioral mechanisms, on the one hand, “directional-motivated reasoning” postulates that partisans tend to base their reasoning on biased sources of information, which leads to inaccurate, desired beliefs reducing cognitive dissonance (Taber and Lodge, 2006; Gaines et al., 2007; Kraft et al., 2015; Miller et al., 2016; Flynn et al., 2017; Peterson and Iyengar, 2020). On the other hand, the “cheerleading effect” posits that when individuals are asked to express their opinions about facts that contrast their political view, they simply ignore these facts and prefer expressing their general affinity toward a specific party or ideology. In this case, partisans are well-informed, but they prefer to express opinions that are in line with their political identity and can contradict the information they have (Bullock et al., 2015; Miller and Conover, 2015; Prior et al., 2015; Schaffner and Luks, 2018; Bullock and Lenz, 2019). For example, Schaffner and Luks (2018) identify the existence of a cheerleading behavior among partisans who express a controversy on the number of people at the 2017 presidential inauguration of Donald Trump and those who participated at Barack Obama's inauguration in 2009. Despite the existence of clear aerial photographs demonstrating that many more people attended Obama's ceremony, a high percentage of Trump voters sustained the opposite.

To study the impact of political narratives on the PGG and the underlying mechanisms behind this effect, we focused on the COVID-19 pandemic and administered a survey experiment in the United States. Given that, since the onset of COVID-19, there is no consensus on its origin, alternative, sometimes competing, narratives about what caused the pandemic have emerged. The main treatments of our survey experiment are built upon two prominent alternative explanations on the origin of the COVID-19 pandemic and are consistent with the concept of narrative outlined by Crow and Jones (2018) and Eliaz and Spiegler (2020). Indeed, each version of the facts represents a “causal model that maps actions into consequences” and contains a cue or a reminder of an existing wider representation of reality that is already part of the public debate. More specifically, the *Lab* narrative, suggests that the pandemic originated as a result of human error and scientific misconduct in laboratories in Wuhan, while the *Nature* narrative describes the biological and genetic origin of the disease without explicitly attributing its cause to human actions. These two narratives have become part of the recent political debate in the United States since the Trump administration sustained the *Lab* narrative on several occasions.^4^ To contrast the diffusion of this narrative, Chinese political representatives and the World Health Organization (WHO) supported the idea that COVID-19 was the result of a natural phenomenon.^5^ Because these narratives entered a political dispute, they both contain political cues that associate them to a specific political party. For instance, a survey conducted in the US from March 10–16, 2020 (Schaeffer, 2020) showed that liberals were more likely than conservatives to state that COVID-19 originated in wildlife (64 vs. 37%). In contrast, conservatives were more likely than liberals to believe that COVID-19 originated in a lab (37 vs. 15%).

These narratives also result in divergent opinions regarding vital policy issues during the post-COVID recovery (Antinyan et al., 2021b). More specifically, individuals in Republican-leaning states voice less favorable opinions about trade openness and the relevance of climate change relative to individuals living in Democratic-leaning states when exposed to the *Lab* narrative.

Regarding the design of the survey experiment, the study participants—individuals residing in the US—were randomly split into three distinct groups: a baseline group involving no narrative manipulation and two treatment groups that were either exposed to the *Lab* or the *Nature* narratives. After the participants had been exposed to the treatment manipulations, we elicited their political preferences. A quick note about the mechanisms through which political narratives affect PGG is worth noting. Unfortunately, our experimental design does not allow us to separate “accuracy-driven reasoning” from “directional-motivated reasoning” since we do not elicit participants' pre-treatment political views. Thus, we use “reasoning effect” to indicate the effects of both the “accuracy-driven reasoning” and “directional-motivated reasoning” on PGG. This means that, in the rest of the paper, we differentiate between the “cheerleading effect” and “reasoning effect.” Nonetheless, although we cannot distinguish ex-ante between the two types of reasoning, we will argue why our experimental results do not support the hypothesis of a gender-specific directionally motivated logic.

The results of our experimental exercise can be summarized as follows. The narratives about the origin of the COVID-19 pandemic increase the PGG. More specifically, relative to a baseline treatment in which no narrative manipulation is implemented, exposing subjects to either the *Lab narrative* or the *Nature narrative* make females more liberal and men more conservative. The PGG effect is amplified by the political orientation of participants' state of residence: the largest gender gap is between men residing in Republican-leaning states and women living in Democratic-leaning states. This result is consistent with the studies arguing that the social context influences how individuals react to political messages and process political information [see, e.g., Martin and Yurukoglu (2017) and Gentzkow et al. (2019)]. Regarding the mechanisms, the cheerleading behavior seems to be the main channel through which narratives contribute to the widening of the PGG. While the literature discusses that the “reasoning effect” and the “cheerleading effect” are not mutually exclusive concepts (Peterson and Iyengar, 2020), we illustrate the prevalence of the latter over the former for the PGG.

The rest of the paper is structured as follows. Section Experimental Design and Data details the experimental design and the data. Section Methodology and Results discusses the empirical methodology and the results. Section Conclusions concludes the paper.

## Experimental Design and Data

### Experimental Design

The survey experiment was run on May 7-8, 2020, through Prolific (Palan and Schitter, 2018), and only US citizens residing in the US were allowed to participate in the study. Three main reasons motivate these participation restrictions. First, citizens are those that have the right to vote, and therefore, it is crucial to understand how political narratives influence the electorate. Second, these restrictions were intended to limit the effects exerted by unobservable social and cultural characteristics of participants. Third, the restrictions reasonably assured that all participants were physically located in the US and were exposed to the same societal, political, and media attention on the COVID-19 pandemic at the time of the experiment.

The survey experiment included three treatments: a baseline *No narrative* treatment and two narrative-manipulated treatments: *Lab narrative* and *Nature narrative*. In all treatments, the questinnaire (see Part E of the Supplementary Information) included several consecutive screens, and each screen contained a single question. After confirming their answer to a question, subjects proceeded to the next screen without having the possibility of moving back to revise previous responses.

The questionnaire administered in the *No narrative* treatment included three main blocks of questions. The first block contained a number of questions to elicit participants' opinions on three relevant policy domains: climate change, foreign trade, and the role of science. The analysis of the answers to these questions (and how they are affected by the narrative manipulations) represents the main research question undertaken in Antinyan et al. (2021b).

The second block contained questions about the potential causes of the COVID-19 pandemic. We used the point allocation method and requested the participants to distribute 100 points across the following four possible causes of the pandemic:

- the virus originated from an accident in a lab;
- the virus originated in nature as a result of natural processes;
- the virus is a weapon the countries use against each other;
- other reasons.

With this question, we aimed at eliciting subjects' beliefs about the real cause of the pandemic: the higher the points allocated to a given cause, the more the subject's belief in the given explanation. The explanation claiming that the virus is a weapon used by some countries against others aimed to distinguish those who believe in a pure conspiracy theory from those who associate the COVID-19 with a lab accident deriving from a human error. If a subject allocated the highest number of points to the fourth explanation, she was requested to indicate the reason she believed had triggered the pandemic.

In the third block, subjects were asked their willingness to get vaccinated against viruses other than COVID-19, their state of residence as well as other socio-demographic questions, including gender, age, occupational and educational status, income situation, whether lockdown restrictions were active in the state where they were actually living, and how much time (in minutes) they spent watching, reading or listening to news about politics and current affairs on a typical day. More importantly, for the scope of the present paper, the third block contained a question asking subjects to report their political view on a 5-point scale, moving from very liberal to very conservative. The political preference question included in our survey experiment is widely used in the literature.

The main difference between the baseline treatment and the narrative-manipulated treatments concerned the fact that, in the latter, before proceeding with the questionnaire, participants were exposed to a specific narrative about the origin of COVID-19. In particular, subjects in the *Lab narrative* treatment were presented with two media extracts claiming that, despite the denials from Chinese authorities, the pandemic was caused by an accident in a laboratory near the wet market in Wuhan. Meanwhile, the two extracts in the *Nature narrative* treatment affirmed that COVID-19 initially originated in the wildlife and then was transmitted to humans presumably from bats and pangolins. Thus, while the *Lab narrative* associates the COVID-19 outbreak with scientific misconduct, the *Nature narrative* emphasizes the importance of science for determining the genetic characteristics of the virus. Furthermore, while the *Nature narrative* depicts the pandemic as a neutral and natural phenomenon, the laboratory narrative attributes the blame to Chinese institutions.

Two aspects of the narrative manipulations implemented in our experiment are worth noting. First, we made sure that each of the narratives was covered by both the democratic leaning and the republican leaning media. In this respect, participants in each of the narrative-manipulated treatments were presented with two extracts, both referring to the same story, but one based on Fox News and one on CNN sources.^6^ Despite the differences in the news networks, the extracts were similar with respect to the framing and wording, and participants were never told the original source the extracts came from. Moreover, while keeping the original text in the extracts mostly unchanged, we simply removed the graphical elements and the precise references to scientific sources (journal articles and names of researchers) to keep the exposition of the two narratives as comparable as possible.

Second, both the stories about the COVID-19 origins circulated in the US debate and media networks before our experiment took place. For instance, on March 17, 2020, Nature Medicine published a scientific article affirming that COVID-19 originated in wildlife. The article represented a scientific reaction to President Trump's rhetoric about the COVID-19 outbreak. On the contrary, on April 15, 2020, Fox News released a report promoting the lab origin of COVID-19. The report gained a lot of media attention throughout the US and triggered a vivid debate in the next days.

To make sure that subjects in the *Lab narrative* and *Nature narrative* treatments fully read and understood the extracts they were exposed to, they were asked to sum up in no more than two sentences what caused the COVID-19 pandemic according to the displayed text. The survey experiment lasted for 5.45 min on average, and the participants were paid £0.84 (around $1.1) for their participation.

### Data

The final sample consists of 3,086 participants: 1,053 in the *No narrative* treatment, 1,016 in the *Lab narrative* treatment, and 1,017 in the *Nature narrative* treatment.^7^ Participants were randomly allocated to one of the three treatments and participated in the study only once. As shown by Part A of the Supplementary Information, the randomization successfully generated balanced subsamples in the three treatments according to the main socio-demographic dimensions. More importantly, for the scope of the paper, in all treatments, participants were equally split between males and females, and the percentage of women was well-balanced across treatments.

Table 1 presents the main descriptive statistics for respondents' socio-demographic characteristics by distinguishing between men and women. The last two columns report the results of a balance test that uses the standardized difference between means and the variance ratio to compare the distributions of men and women's characteristics. Although there is no clear threshold of these two statistics to define imbalance, Rubin (2001) suggests a cut-off in the standardized difference of 0.25 and a variance ratio between 0.5 and 2.^8^ In general, we may say that standardized differences (variance ratios) should be as close to zero (one) as possible.

**Table 1:** Summary statistics for socio-demographic characteristics.

	**Male (*****N*** **=** **1,509)**		**Women (*****N*** **=** **1,577)**		**Balance**	
	**Mean**	**Variance**	**Mean**	**Variance**	**Std-diff**	**Var-ratio**
Age	33.976	154.700	35.221	175.766	−0.097	0.880
Income	6.155	3.231	5.982	3.281	0.096	0.985
Republican state (rep)	0.338	0.224	0.354	0.229	−0.033	0.979
COVID-19	0.099	0.016	0.094	0.014	0.039	1.099
Lockdown	0.782	0.171	0.774	0.175	0.019	0.975
Lower than high school	0.008	0.008	0.008	0.008	0.004	1.045
High school	0.346	0.226	0.342	0.225	0.007	1.005
Bachelor's degree	0.455	0.248	0.469	0.249	−0.028	0.996
Master's degree	0.140	0.121	0.139	0.120	0.005	1.010
Doctoral degree	0.050	0.048	0.042	0.040	0.041	1.193
Employed	0.577	0.244	0.467	0.249	0.221	0.981
Self-employed	0.102	0.092	0.124	0.108	−0.068	0.846
Student	0.161	0.135	0.164	0.137	−0.009	0.984
Unemployed	0.129	0.112	0.190	0.154	−0.167	0.729
Other	0.032	0.031	0.056	0.053	−0.117	0.585
Metro county	0.867	0.115	0.837	0.136	0.086	0.843
Republican county	1.353	0.229	1.403	0.241	−0.102	0.950
Marriage rate	6.095	4.209	6.162	5.099	−0.031	0.825

*This table reports the main descriptive statistics for men and women's socio-demographic characteristics. The last two column provides two distributional tests aiming to check whether the two groups are balanced in terms of these characteristics. Std-diff is the standardized difference between the means, while Var-ratio is the corresponding variance ratio [see Linden and Samuels (2013)]*.

The average age of men is 33.976 years, while for women, this average is 35.221 years. However, no significant differences emerge in terms of age between the two groups. Even the distribution of self-reported income is similar between men and women. Here, participants were asked to indicate their income status using a scale on which 1 was the lowest income group and 10 the highest income group in the United States. This variable is particularly important to control for the economic factors mentioned in the Introduction that can potentially affect women's preferences toward a larger welfare state (note that one of the main explanations of the PGG relies on preferences about the welfare state).^9^

No difference between men and women emerges in terms of variable *rep*: a dummy which equals 1 if the respondent resides in a Republican-leaning state and 0 otherwise. We classified states using the average party affiliation of each state's residents throughout 2018.^10^ The variable COVID-19 represents the COVID-19 incidence rate measured as the ratio between the cumulated number of COVID-19 cases officially confirmed in each state till the day before the survey experiment and the corresponding population (USA Facts, 2020). Approximately 78 percent of men and women reported to live in a location subject to lockdown restrictions at the time of the survey. This variable captures subjects' perception to live under restrictions and thus their political view. Nonetheless, to check the robustness of our results, in Part C of the Supplementary Information, we repeat our main analysis by replacing individual perception with official information on state restrictions.

Men and women are also homogeneous in terms of educational levels. Indeed, there are no significant discrepancies between the two groups across the four classes of educational attainment: lower than high school, high school, bachelor's degree, master's degree, and doctoral degree. In contrast, small differences between the two sexes arise when we look at the occupational status. In particular, men are more likely to be employed and less likely to be unemployed than women. In order to control for the socio-political environment in which subjects live, we supplement data with information on whether they reside in metropolitan areas characterized by more than 250,000 inhabitants and in republican counties. We identified counties' political orientation using the average vote share for the democratic or republican candidate in the last five presidential elections run before the experiment.^11^ Finally, we also included the state marriage rates per 1,000 total population provided by the National Center for Health Statistics (NCHS).^12^ Although we already control for individual income that has been associated with relative preferences for the welfare state and hence the PGG, the inclusion of the state marriage rates allows us to control for other social determinants of women's conditions such as more favorable state legislation or better economic opportunities.

## Methodology and Results

### Methodology

This study investigates whether narratives on COVID-19 origins affect the Partisan Gender Gap. Since the dependent variable classifies individual political preferences into five classes of conservativism, we consider both a linear specification and an ordered probit model.^13^ The five classes used to measure individual preferences are: 1 = very liberal, 2 = liberal, 3 = moderate, 4 = conservative, 5 = very conservative.

Denoting with *T* = *B, L, N* the *Baseline* treatment, *Lab narrative*, and *Nature narrative*, respectively, we start by running the following OLS regression:

(1) $$\begin{array}{l} PV_{i}=\alpha +\beta \cdot male_{i}+\gamma \cdot rep_{i}+\delta \cdot T_{i}+\mu \cdot T_{i}\cdot male_{i}\\ \;+\rho \cdot T_{i}\cdot rep_{i}+\phi^{\prime }X_{i}+\varepsilon_{i}, \end{array}$$

where *PV*_*i*_ is the political view of individual *i*, *T*_*i*_ is the treatment individual *i* was assigned to (the *Baseline* treatment is the omitted group), *male*_*i*_ is a dummy variable taking value 1 if the respondent declares to be a man and zero otherwise, *rep*_*i*_ is a dummy taking value 1 if the respondent lives in a Republican-oriented state and zero otherwise, *X*_*i*_ is a set of control variables describing individual socio-demographic characteristics, and ε_*i*_ is the error term. The inference is based on heteroskedasticity-robust standard errors. Because Antinyan et al. (2021a) found that the state political orientation moderates the effect of narratives on political preferences, we estimate Equation (1) with and without the following constraint: ρ = 0 for any treatment.^14^

Table 2 summarizes the expected outcomes from Equation (1) for respondents with the various combinations of treatment, gender, and state political orientation (the residual variation term is omitted).

**Table 2:** Expected outcomes.

**Treatment**	**Gender**	**State**	**Expected outcome**
Baseline	Female	Dem	$\alpha +\phi^{\prime }{\bar{X}}_{i}$
Baseline	Female	Rep	$\alpha +\gamma +\phi^{\prime }{\bar{X}}_{i}$
Baseline	Male	Dem	$\alpha +\beta +\phi^{\prime }{\bar{X}}_{i}$
Baseline	Male	Rep	$\alpha +\beta +\gamma +\phi^{\prime }{\bar{X}}_{i}$
Narrative	Female	Dem	$\alpha +\delta +\phi^{\prime }{\bar{X}}_{i}$
Narrative	Female	Rep	$\alpha +\gamma +\delta +\rho +\phi^{\prime }{\bar{X}}_{i}$
Narrative	Male	Dem	$\alpha +\beta +\delta +\mu +\phi^{\prime }{\bar{X}}_{i}$
Narrative	Male	Rep	$\alpha +\beta +\gamma +\delta +\mu +\rho +\phi^{\prime }{\bar{X}}_{i}$

From Table 2, we can easily derive the average treatment effect of treatment T on the Partisan Gender Gap:

(2) $$\begin{array}{l} \Delta PGG\equiv \left(\;\bar{PV}{|}_{T=L,N,male=1}-\;\bar{PV}{|}_{T=L,N,male=0}\right)\\ \;-\left(\;\bar{PV}{|}_{T=B,male=1}-\;\bar{PV}{|}_{T=B,male=0}\right)=\mu . \end{array}$$

Thus, a positive value of μ indicates that, compared to the baseline group, narrative *T*=*L, N* enlarges the PGG, whereas a negative value would denote a shrinking effect. Moreover, Equation (1) allows us to distinguish the political view of men and women living in Democratic- and Republican-leaning states.

Because of the discrete nature of our dependent variable, we also estimate an ordered probit model. Formally, we estimate the probability of declaring a political view equal to *k* as follows:

(3) $$\begin{array}{l} \mathrm{Pr}\left[PV_{i}=k\right]=F\left(z_{k}-{\text{W}}_{\text{i}}w\right)-F\left(z_{k-1}-{\text{W}}_{\text{i}}w\right), \end{array}$$

where **W**_**i**_*w* is the right-hand side of Equation (1) with the exclusion of the error term, *F*(•) is the standard normal cumulative distribution function, and *z*_*k*_ is the cut point of class *k*.

**Figure 1:**
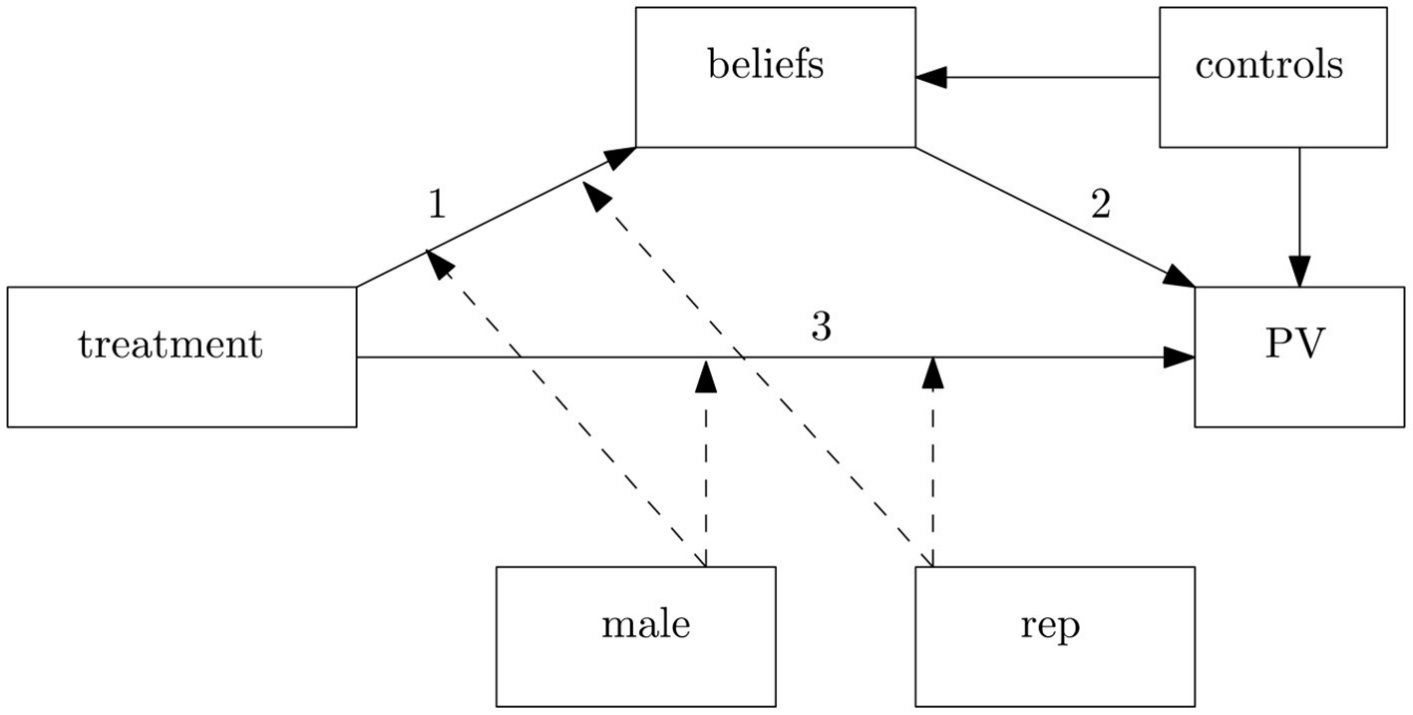
Path diagram for SEM decomposition. This diagram indicates how narratives, moderators (male and rep), and control variables enter our structural equation model (SEM). Solid lines denote the estimated direct relationship between two variables, whereas the dashed lines indicate the presence of interaction effects. Edges 1 and 2 characterize the indirect (reasoning) effect of narratives on political views (PV). In contrast, edge 3 represents the direct (cheerleading) effect of narratives on PV.

The last part of the analysis exploits the question regarding respondents' beliefs on COVID-19 causes. Following Antinyan et al. (2021a), we use a Structural Equation Model (SEM) to decompose our estimates into two components: the reasoning effect and the cheerleading effect. The reasoning effect is the part of the total effect passing through individual beliefs about COVID-19 causes. In contrast, the cheerleading effect is the part of the total effect unexplained by these beliefs. Figure 1 shows the path diagram associated with our SEM. Here, we can identify two distinct channels linking our treatments with political view (PV). The first channel represents the reasoning effect and includes links 1 and 2. According to this channel, narratives can influence personal opinions about what generated the COVID-19 (link 1), and these beliefs may affect individual preferences (link 2). Because we observe only the post-treatment political view, we cannot say whether narratives lead to politically biased or unbiased reasoning, so we cannot distinguish between accurate or directionally motivated reasoning. However, if this channel yields statistically significant results, we may conclude that narratives influence political views through a cognitive process. In contrast, the second channel is not mediated by beliefs (link 3) and represents a pure cheerleading effect. Notice that, in line with Equation (1), we allow the *male* and *rep* dummy to moderate both channels (see the dashed links in Figure 1).

The path diagram represented in Figure 1 can be expressed in terms of structural equations as follows:

(4) $$\begin{array}{l} B_{i}^{c}=a+b\cdot male_{i}+q\cdot rep_{i}+d\cdot T_{i}+m\cdot T_{i}\cdot male_{i}\\ \;+r\cdot T_{i}\cdot rep_{i}+p^{\prime }X_{i}+e_{i}, \end{array}$$

and

(5) $$\begin{array}{l} PV_{i}=\alpha +\beta \cdot male_{i}+\gamma \cdot rep_{i}+\delta \cdot T_{i}+\mu \cdot T_{i}\cdot male_{i}\\ \;+\rho \cdot T_{i}\cdot rep_{i}+\phi^{\prime }X_{i}+\underset{c}{\sum }\sigma_{c}\cdot B_{i}^{c}+\varepsilon_{i}, \end{array}$$

where $B_{i}^{c}$ is the number of points that subject *i* assigned to cause *c* and represents his/her beliefs. Using the terminology adopted in mediation analysis, we can refer to Equation (4) as “mediation equation,” whereas Equation (5) is typically called “outcome equation.” Since our survey considered different potential causes of COVID-19, each cause will have its mediation equation. Because beliefs about COVID-19 origins (i.e., mediators) are correlated, we allow residuals of the mediators to be correlated.

To measure the indirect effect of narratives (as well as of any other covariate) on subjects' political view, we must multiply the coefficients estimated in Equation (4) by the coefficient of beliefs estimated in Equation (5) (i.e., σ_*c*_ for any hypothesized cause *c*) and taking their sum. In other words, for treated subjects, the treatment effect passing through individual beliefs will be given by $\underset{c}{\sum }\sigma_{c}\cdot d$. In this way, we capture any rational or motivated reasoning effect of a narrative passing through individual beliefs on COVID-19 causes (links 1 and 2 in Figure 1). Analogously, the indirect effect of any other control variable such as *rep*_*i*_ or *X*_*i*_ will be given by $\underset{c}{\sum }\sigma_{c}\cdot q$ or $\underset{c}{\sum }\sigma_{c}\cdot p$. In contrast, the coefficients in Equation (5) measure the direct impact of explanatory variables on political views and capture the cheerleading effect, that is, the effect that is not mediated by any rational or motivated reasoning on COVID-19 causes (link 3 in Figure 1). We use the delta method to compute the standard errors of both the cheerleading and the reasoning effect.^15^

### Results

**Figure 2:**
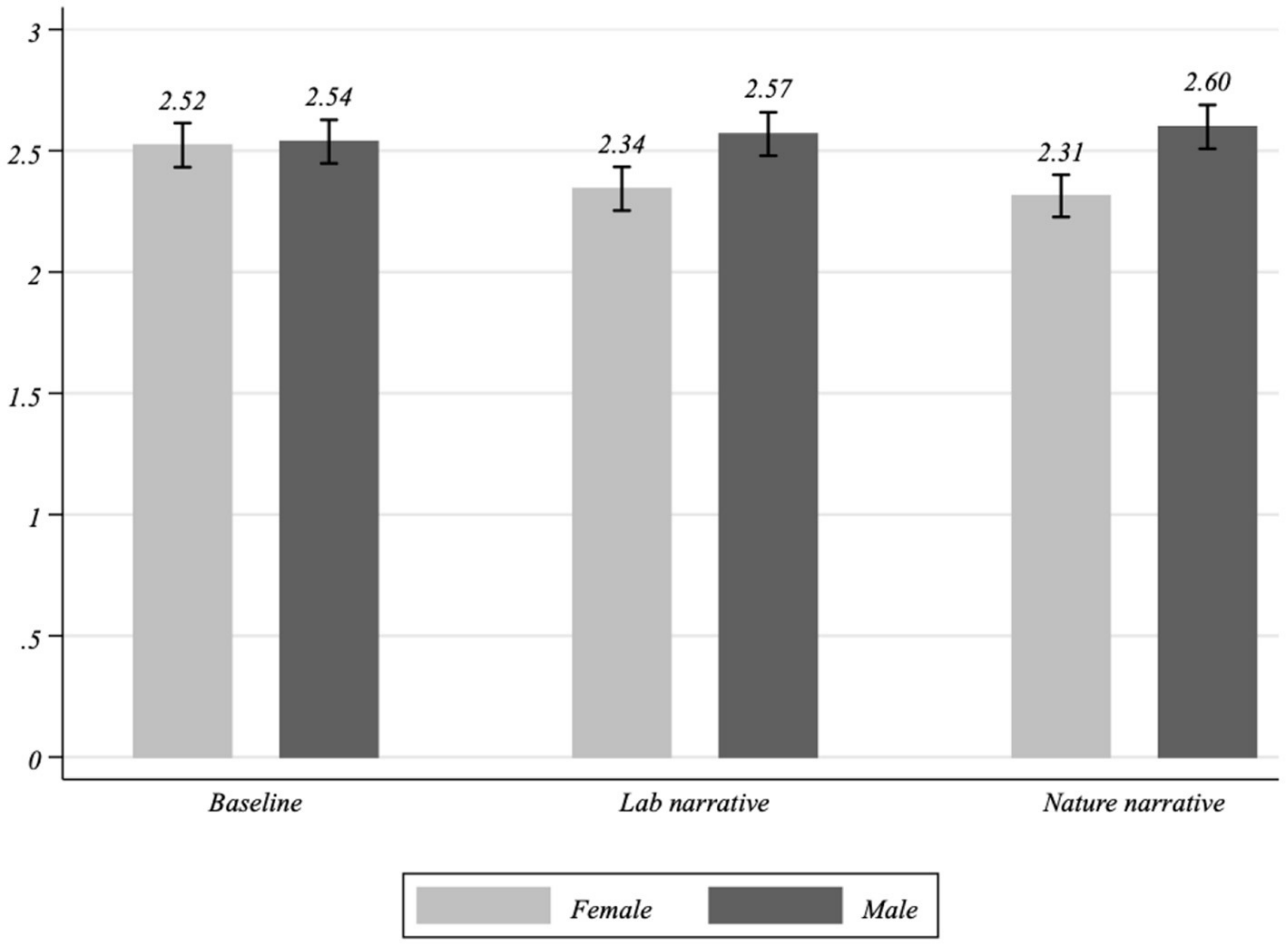
Political views by gender and treatment. Histograms indicate the unconditional average political view of men and women subject to different treatments. The corresponding 95% confidence intervals are represented with capped spikes.

Figure 2 displays the average political view declared by men and women across different treatment groups. Whereas there are no differences between male and female preferences in the baseline group, significant differences seem to emerge for men and women treated with the two narratives. In particular, treated women declare more liberal views, whereas treated men report more conservative positions. We used a Kruskal-Wallis test to support visual interpretation. According to this test, the difference between genders is statistically insignificant in the baseline group [χ^2^(1) is 0.295 with *p* = 0.587]. In contrast, a Partisan Gender Gap seems to appear when subjects are treated with narratives (χ^2^(1) is 11.628 with *p* = 0.001 for the *Lab narrative*, and χ^2^(1) 21.419 with *p* = 0.000 for the *Nature narrative*).

Table 3 reports our main results. Columns 1 and 2 show the OLS estimates of Equation (1), while Columns 3 and 4 provide the corresponding Ordered Probit estimates. In Columns 1 and 3, we interacted the factor variable indicating the treatment group with the *male* dummy, assuming ρ = 0 for any narrative. The positive coefficients of the interaction terms (i.e., Lab narrative^*^male and Nature narrative^*^male) indicate that the distance between men's political positions and women's political positions widens once subjects are treated with one of the two narratives. In other words, whereas the treated women tend to declare more liberal positions compared to the control treatment, the treated men do not change their political position compared to the control which enlarges the PGG. This evidence is in line with Figure 2.

**Table 3:** Political view (OLS and ordered probit).

	**OLS**		**Ordered probit**	
	**(1)**	**(2)**	**(3)**	**(4)**
Lab narrative	−0.154^**^	−0.241^***^	−0.169^**^	−0.257^***^
	(0.064)	(0.071)	(0.067)	(0.075)
Nature narrative	−0.165^***^	−0.229^***^	−0.176^***^	−0.242^***^
	(0.062)	(0.070)	(0.066)	(0.074)
Lab narrative*male	0.183^**^	0.186^**^	0.201^**^	0.204^**^
	(0.090)	(0.090)	(0.093)	(0.093)
Nature narrative*male	0.232^***^	0.235^***^	0.248^***^	0.251^***^
	(0.089)	(0.089)	(0.093)	(0.093)
Male	0.045	0.042	0.044	0.041
	(0.063)	(0.063)	(0.065)	(0.065)
Rep	0.092^**^	−0.042	0.091^**^	−0.046
	(0.045)	(0.070)	(0.046)	(0.072)
Lab narrative*rep		0.242^**^		0.243^**^
		(0.097)		(0.100)
Nature narrative*rep		0.171^*^		0.177^*^
		(0.095)		(0.098)
Constant	2.124^***^	2.196^***^		
	(0.183)	(0.185)		
Additional controls	Yes	Yes	Yes	Yes
Observations	3,086	3,086	3,086	3,086
*R*^2^ and Pseudo-*R*^2^	0.061	0.063	0.021	0.022
Log-likelihood			−4290.107	−4286.770
*F*-statistics/Wald χ^2^ for nested models	10.97	2.60	183.46	4.38
DF	18	2	18	2
*P*-value (for nested models)	0.000	0.074	0.000	0.112

*Coefficients of Equations (1) and (3). Additional controls include respondent's education level, an indicator variable for individuals living under lockdown restrictions, a dummy for those who live in metro areas of more than 250,000 population, a dummy for respondents living in Republican-leaning counties, a self-reported assessment of personal income, respondent's age, and employment status. Finally, we also included the COVID-19 incidence rate recorded in the respondent's state till the day before the interview and the state marriage rate. The complete set of estimates is available in the Part F of the Supplementary Information. Robust standard errors in parentheses*.

*Significance levels:*

* *p < 0.1*,

** *p < 0.05*,

*** *p < 0.01*.

In Columns 2 and 4, we also interact the treatment indicator with the dummy variable indicating Republican-leaning states. This allows us to take into account the fact that narratives on COVID-19 origins cause political polarization between subjects living in Democratic-leaning and Republican-leaning states (Antinyan et al., 2021a). The *F*-test and Wald-χ^2^ test reported at the end of Columns 2 and 4, respectively, indicate that the inclusion of this second interaction term slightly improves the model specification. As before, coefficient μ is positive for both narratives, which implies that treated subjects continue to exhibit a PGG.^16^ Because PV has a standard deviation of 1.04, the interpretation of OLS coefficients reported in Table 3 and of our results in general are rather straightforward. In particular, a single exposure to the Lab narrative induces a PGG of 17.9 percentage points of standard deviation (i.e., 0.186/1.04), whereas a single exposure to the Lab narrative induces a PGG of 22.6 percentage points of standard deviation (i.e., 0.235/1.04).

Notice that, when controlling for socio-demographic characteristics (such as income, education, and social context in which participants live) that have been used to explain the PGG (Kaufmann, 2002; Huddy et al., 2008), we find no difference in political positions between women and men assigned to the baseline group.^17^ Interestingly, we would expect to observe PGG in the baseline group, nonetheless its absence can be due to the characteristics of the subject pool that participates in the on-line experiment. Indeed, while online experiments permit us to recruit a broader population than classical lab experiments with students, internet and platform users can still be different from the population at large (Palan and Schitter, 2018, Coppock, 2019). Please note that the absence of the PGG in the baseline group, does not harm the internal validity of the study, since the randomization is successful and the subjects in the three treatment arms possess similar characteristics. Thus, the increase of the PGG in the treatment groups can be attributed to the narratives the subjects are exposed to with very high confidence. Commenting on the external validity of the results, we think that the political narratives studied can make the PGG even wider at the population level, where socio-demographic differences between men and women exist.

**Figure 3:**
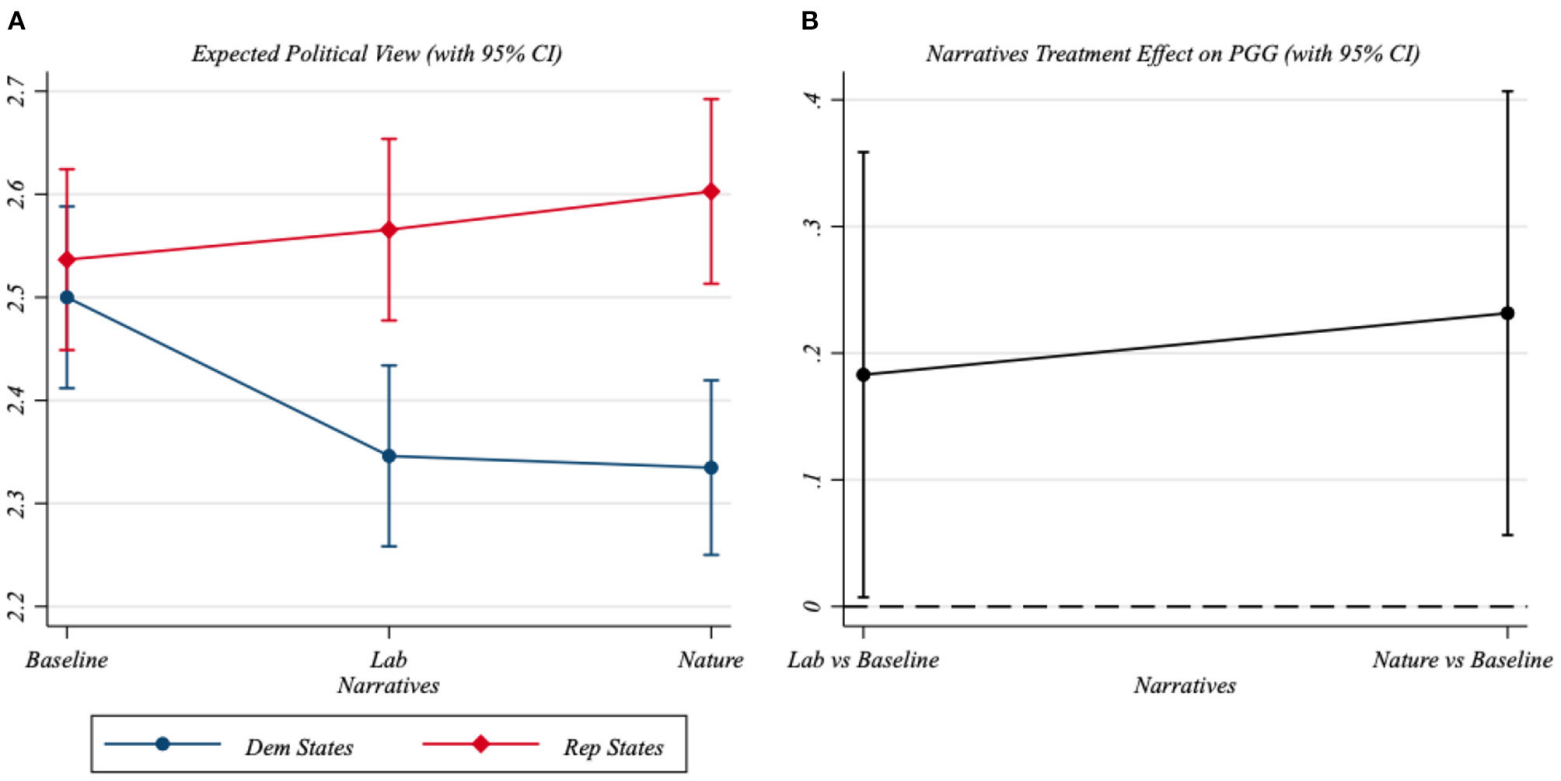
Heterogeneous treatment effects and Partisan Gender Gap. This figure is based on the results reported in Column 2 of Table 3. The left panel shows the predicted political views by gender and treatment status. The right panel contrasts the gender gap for treated participants with the gender gap for the baseline group. The corresponding 95% confidence intervals are represented with capped spikes. **(A)** Expected political view (with 95% CI). **(B)** Narratives treatment effect on PGG (with 95% CI).

Figure 3 provides a graphical representation of results reported in Column 2 of Table 3. In particular, panel A displays the expected political view (with the 95% CI) of men and women separately. Notice that, whereas no significant differences emerge between men and women in the baseline group, the distance between men and women's positions enlarges when respondents are treated with one of the two narratives. More specifically, treated women tend to declare more liberal views. Panel B reports the difference between the PGG for treated subjects and the PGG for subjects in the baseline group. This figure shows that the Partisan Gender Gap in the treated groups is significantly higher than the gender gap in the baseline group.

***Result 1***: Political narratives on the origins of COVID-19 increase the PGG by pushing women toward more liberal positions.

**Figure 4:**
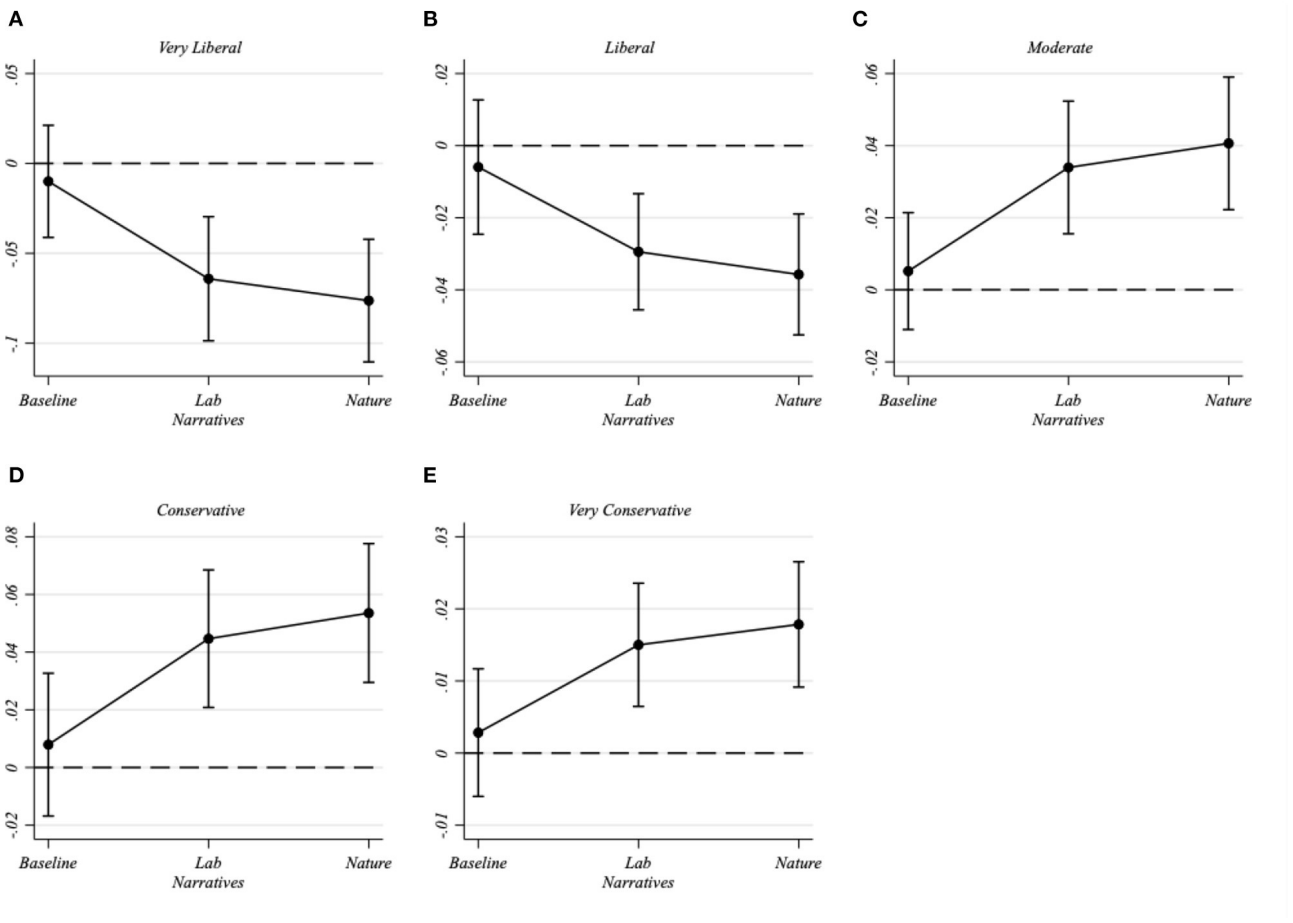
Gender Gap for different political positions. Based on the results reported in Column 4 of Table 3, this figure shows the differences between men's and women's predicted probabilities to declare a specific political position across treatments. In each panel, positive (negative) values indicate that men are more likely than women to declare that political view. The corresponding 95% confidence intervals are represented with capped spikes. **(A)** Very liberal. **(B)** Liberal. **(C)** Moderate. **(D)** Conservative. **(E)** Very conservative.

Using the Ordered Probit estimates reported in Column 4 of Table 3, Figure 4 illustrates the probability gap between men and women expressing a specific political position across different treatments (i.e., the difference between the probability that men have a political view equal to *k* and the probability that women have the same view). Panels A and B reveal that, in the treatment groups, women are more likely than men to declare liberal and very liberal preferences compared to the baseline group. For instance, the probability that women treated with the Lab narrative express very liberal positions is more than 5 percentage points higher than the probability that men exposed to the same treatment declare very liberal positions (Panel A). In contrast, treated men are more likely than treated women to express moderate, conservative, or very conservative positions (panels C, D, and E). By looking at Panel D, we may notice that the probability that men exposed to the Lab (Nature) narrative declare a conservative view is about 4 (6) percent higher than the probability that women treated with the same narrative express the same position.

**Figure 5:**
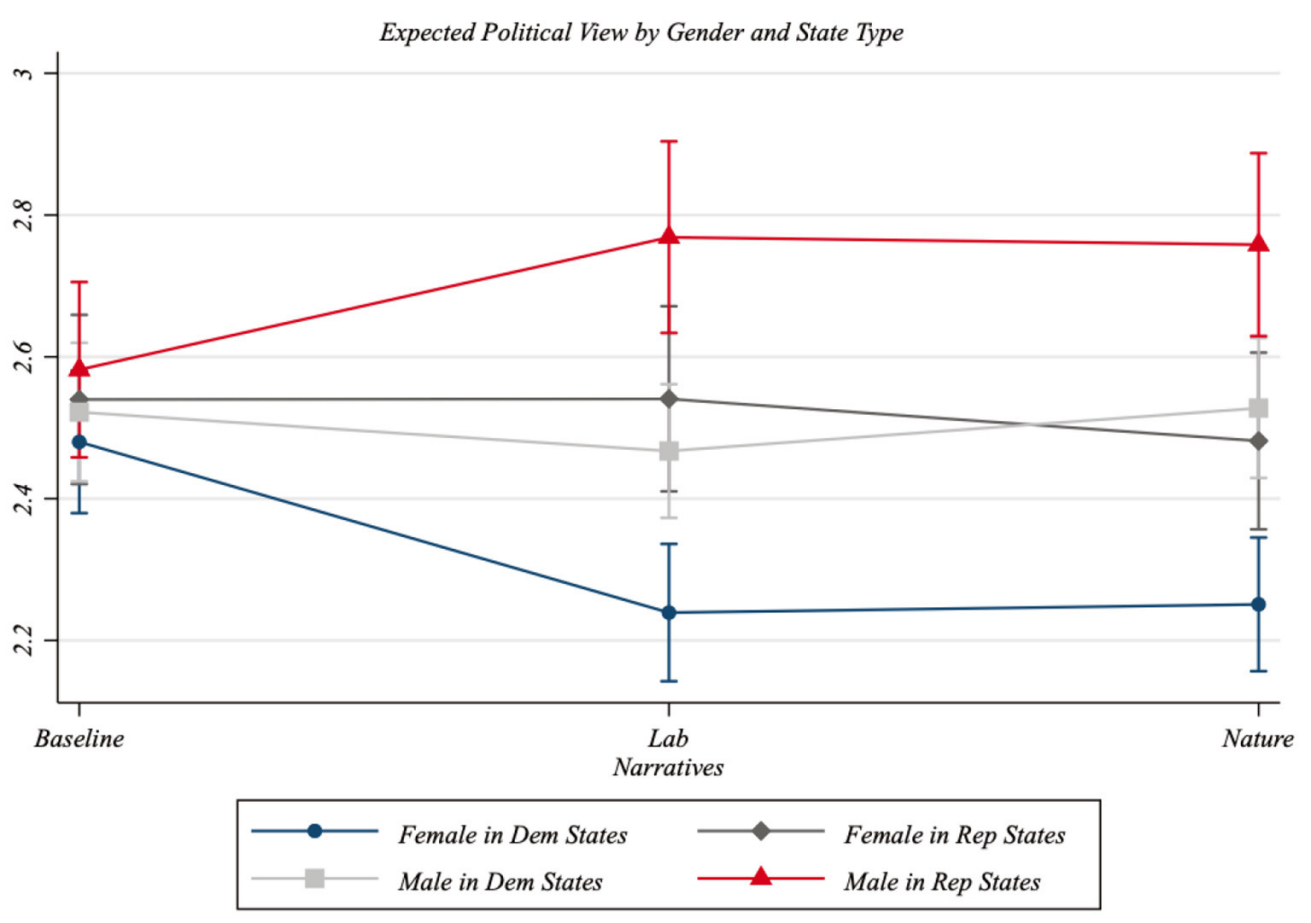
Partisan Gender Gap and state type. Based on the results reported in Column 2 of Table 3, this figure shows the predicted political views by gender and treatment status, distinguishing between individuals living in Republican-leaning states and those living in Democratic-leaning states. The corresponding 95% confidence intervals are represented with capped spikes.

Column 2 of Table 3 also allows us to distinguish the PGG across state types. Therefore, we also computed the expected political view of men and women living in Republican- and Democratic-leaning states separately. Figure 5 indicates that the largest PGG is between men residing in Republican-leaning states and women living in Democratic-leaning states. Indeed, adding the 17.9% points of PV standard deviation due to the Lab narrative's PGG effect to the 23.2% points associated with state differences, we obtain a distance between men residing in Republican-leaning states and women living in Democratic-leaning states of more than 40 standard deviation points.

***Result 2***: State political differences magnify the effect of narratives regarding the origins of COVID-19 on the gender gap in political views.

In Table 4, we decompose the total effects reported in Column 2 of Table 3 into the cheerleading and the reasoning effect.^18^ According to our results, women exhibit both components (see the coefficients of *Lab narrative* and *Nature narrative*). In the *Lab narrative* case, these two components have opposite effects, and the cheerleading effect dominates the reasoning one. In the *Nature narrative* case, both the cheerleading and the reasoning effects push women toward more liberal positions. Notice that, although we cannot distinguish between accuracy-driven and directionally motivated reasoning, we can conclude that treated subjects' reasoning is not directionally motivated. Indeed, independently of whether partisans react to political cues with directionally motivated reasoning or a cheerleading behavior, these two effects should always exhibit the same sign, causing more political polarization. Therefore, given that men and women do not differ in terms of reasoning, we can indirectly infer that both sexes adopt an accuracy driven logic, but this logic is masked by a cheerleading behavior.

**Table 4:** Political view (decomposition).

	**Cheerleading**	**Reasoning**	**Total**
	**(1)**	**(2)**	**(3)**
Nature hypothesis			−0.005^***^
			(0.001)
Accident hypothesis			0.010^***^
			(0.001)
Weapon hypothesis			0.007^***^
			(0.001)
Lab narrative	−0.360^***^	0.119^***^	−0.241^***^
	(0.065)	(0.030)	(0.071)
Nature narrative	−0.137^**^	−0.092^***^	−0.229^***^
	(0.064)	(0.030)	(0.070)
Lab narrative*male	0.194^**^	−0.008	0.186^**^
	(0.081)	(0.037)	(0.089)
Nature narrative*male	0.197^**^	0.038	0.235^***^
	(0.081)	(0.037)	(0.089)
Male	0.084	−0.042	0.042
	(0.057)	(0.026)	(0.063)
Rep	−0.035	−0.007	−0.042
	(0.062)	(0.028)	(0.068)
Lab narrative*rep	0.199^**^	0.043	0.242^***^
	(0.085)	(0.039)	(0.093)
Nature narrative*rep	0.182^**^	−0.011	0.171^*^
	(0.085)	(0.039)	(0.093)
Additional controls	Yes	Yes	Yes
Observations	3,086	3,086	3,086
*R*^2^	0.224		0.166

*This table reports the cheerleading and reasoning effects estimated through the structural equation model described by Equations (4) and (5). Column 1 provides the cheerleading effect (i.e., each covariate's direct effect on individual political views). These coefficients correspond to the estimates of Equation (5). For each covariate, Column 2 reports the reasoning effect (i.e., the effect of a covariate on political view passing through individual beliefs on COVID-19 causes). These effects are computed by multiplying the coefficient in Equation (4) with the estimated coefficients of COVID-19 causes in Equation (5). Column 3 gives the total effect of two components (i.e., the sum of direct and indirect effects). The other remarks about the additional controls of Table 3 apply. Standard errors are in parentheses and covariance among equations is allowed*.

*Significance levels*:

* *p < 0.1*,

** *p < 0.05*,

*** *p < 0.01*.

By looking at the coefficients of Lab narrative^*^male and Nature narrative^*^male, we can say that men exhibit a lower cheerleading component, while the reasoning effect remains unchanged.

Finally, in line with Antinyan et al. (2021a), we found that subjects residing in Republican-leaning states react to our narratives with a cheerleading behavior that pushes them to declare more conservative positions (see the coefficients of Lab narrative^*^rep and Nature narrative^*^rep).

***Result 3***: The PGG arising from exposing subjects to narratives on COVID-19 origins is the consequence of a cheerleading behavior.

## Conclusions

In this paper, we explored how men and women respond differently to political narratives. In particular, we examined the effect of narratives on the origins of COVID-19 on the US Partisan Gender Gap, that is, the increasing political gap between males and females. To do this, we randomly assigned subjects to three different treatments: a non-narrative treatment (the baseline), a treatment ascribing the cause of the COVID-19 to a human error that occurred in a Chinese lab (*Lab narrative*), and a treatment suggesting that COVID-19 is a natural phenomenon originating from wildlife (*Nature narrative*). These two narratives were already circulating in the US before our experiment, so subjects had the opportunity to locate them within the political debate. Indeed, the *Lab narrative* has been supported by Trump's administration on several occasions, whereas the *Nature narrative* represented the main opposing narrative to Trump's rhetoric. This means that both stories were potentially associated with some political cues.

We found that these cues were strong enough to push women toward more liberal positions, enhancing the Partisan Gender Gap. Both narratives are particularly effective in activating women living in Democratic-leaning states and men residing in Republican-leaning states. Compared to the baseline group, the former tended to declare more liberal positions, whereas the latter responded by adopting a more conservative view. Finally, we investigate whether the polarizing effect of narratives passes through reasoning or is the consequence of a pure cheerleading behavior. We find that reason plays the same role in both sexes; thus, the Partisan Gender Gap increases because of cheerleading behaviors. In other words, males and females react to narratives on COVID-19 causes reinforcing or reaffirming their political identity, mainly when this identity is supported by the socio-political context in which they live.

These results suggest that attaching political cues to pieces of information that could otherwise be decision-relevant, may favor partisan affiliation to become the salient dimension, amplifying the partisan gender gap.

## Data Availability Statement

The raw data supporting the conclusions of this article will be made available by the authors, without undue reservation.

## Ethics Statement

The studies involving human participants were reviewed and approved by the Ethical Committee of the University of Venice Ca' Foscari. The patients/participants provided their written informed consent to participate in this study.

## Author Contributions

All authors listed have made a substantial, direct and intellectual contribution to the work, and approved it for publication.

## Conflict of Interest

The authors declare that the research was conducted in the absence of any commercial or financial relationships that could be construed as a potential conflict of interest.

## Footnotes

^1^In general, we can define partisanship as “an individual's adherence to a particular political party or platform” (Klar, 2014, p. 687). Two essential features of partisanship are that it tends to remain highly stable over time and influences individual perceptions of political cues (Bartels, 2002), where political cues are explicit or implicit signals indicating which ideological group supports a specific stance.

^2^According to Shapiro and Mahajan (1986), men exhibit a higher degree of conservativism than women in policy issues such as criminal justice, national defense, law enforcement, and the welfare state. In line with these results, Kaufmann and Petrocik (1999) find that men prefer lower welfare spending than women.

^3^Using a panel data approach, Edlund and Pande (2002) explain the fact that in the last decades, more women than men voted for the Democratic party with the decline in marriage. Edlund and Pande argue that divorce makes women economically vulnerable, shaping their political preferences in favor of liberal positions that are typically associated with higher spending in the welfare state. Other studies reaching similar conclusions are Box-Steffensmeier et al. (2004) and Iversen and Rosenbluth (2006).

^4^Dan Mangan and Berkeley Lovelace Jr., “Trump suspects coronavirus outbreak came from China lab, doesn't cite evidence,” CNBC, www.cnbc.com/2020/04/30/coronavirus-trump-suspects-covid-19-came-from-china-lab.html (accessed April 30, 2020).

^5^Cecelia Smith-Schoenwalder, “WHO Assures That Coronavirus Is Natural Amid Trump Attacks,” US News, www.usnews.com/news/world-report/articles/2020-05-01/who-assures-that-coronavirus-is-natural-amid-trump-attacks (accessed May 1, 2020). Meg Kelly and Sarah Cahlan, “Was the new coronavirus accidentally released from a Wuhan lab? It's doubtful,” The Washington Post, www.washingtonpost.com/politics/2020/05/01/was-new-coronavirus-accidentally-released-wuhan-lab-its-doubtful (accessed May 1, 2020).

^6^Three (out of four) of the extracts were taken directly from the websites of the two media outlets, while the *Nature narrative* associated with a conservative media network was taken from the Daily Caller, a source that is, however, directly connected to Fox News (the owner of Daily Caller was Tucker Carlson at the time the survey experiment was carried out, one of the most influent anchormen of Fox News.

^7^Out of the initial 3,091 participants, we excluded five participants who completed the questionnaire but either refused to provide demographic information or gave insensate responses: two subjects refused to provide their age, whereas three persons reported inexistent states of residence. The attrition bias referring to those that entered the survey and quit before the end of the questionnaire is 2.71 percent (i.e., 84 questionnaires out of 3,091). Because the tasks are usually short and the subjects get paid for their participation, the attrition rate in Prolific is usually rather low (Palan and Schitter, 2018).

^8^Normand et al. (2001) consider a standardized difference greater than 0.10 as indicative of imbalance. Because the standardized difference is a version of Cohen's d statistic for effect size (Cohen, 1988), a difference of 0.2 represents a “small” discrepancy.

^9^One can argue that a self-reported status is less reliable than an objective measure of income. However, as shown in Karadja et al. (2017), individual preferences for redistributive policies depend more on the self-perceived relative status than on effective income. Moreover, different groups of individuals might be differently equipped to provide their income based on a specific time horizon (e.g., annual, monthly, weekly, etc.). In contrast, a ten-point scale does not depend on any given periodicity. Finally, people are sensitive about disclosing their income, while a scale can make people feel more comfortable about sharing information.

^10^Data comes from Jones (2019). Two important features characterize Jones' data: they include nonvoters' political position, and all measures refer to the same period. We consider as Republicans those states with a fraction of affiliations greater or equal to the fraction of Democrats. In Table C3 of the Supplementary Information, we conduct a robustness check, separating blue/red states from those that changed their political orientation in the 2020 US Presidential Elections. Nonetheless, our results continue to hold.

^11^To distinguish between metro and non-metro counties, we used the “2013 Rural-Urban Continuum Codes” provided by the U.S. Department of Agriculture (available at https://www.ers.usda.gov/data-products/rural-urban-continuum-codes.aspx, accessed 15 June 2020). Data on presidential elections come from the “County Presidential Election Returns 2000-2016” provided by the MIT Election Data and Science Lab in 2018 (available at https://electionlab.mit.edu/data, accessed 15 June 2020).

^12^Data on marriage rates are available at https://www.cdc.gov/nchs/fastats/marriage-divorce.htm (accessed 10 April 2021).

^13^We also carry out an ordered logit analysis and a Brant test for the parallel odds assumption in Part C of the Supplmentary Information.

^14^We use an *F*-test (Wald-χ^2^ test in case of ordered probit models) to check whether this restriction is appropriate as well as to verify the opportunity to include a three-way interaction term among treatment, gender, and state political orientation.

^15^See MacKinnon et al. (2007) for further methodological details on mediation analysis.

^16^We have also considered a three-way interaction model in which narratives are interacted with state political orientation and gender. However, with a *p*-value of 0.321 and 0.391, respectively, the *F*-test and Wald-χ^2^ test for nested specifications reveal that a three-way interaction model does not significantly improve the reduced model estimated in Columns 2 and 4. Therefore, we opted for more parsimonious specifications, such as those reported in Table 2.

^17^The coefficients of control variables indicate that individual income, education, and county-level political orientation influence political preferences (see Table F1 of the Supplementary Information). This implies that, in a more heterogeneous population, such factors might also cause a PGG in the baseline group that is not related to narratives.

^18^Table 3 only shows the estimates of Equation (5). The coefficients of the mediation equations, represented by Equation (4), are in Table B1 of the Supplementary Information.

## References

[B1] Andreoni J. Mylovanov T. (2012). Diverging opinions. Am. Econ. J. Microecon. 4, 209–232. 10.1257/mic.4.1.209

[B2] Antinyan A. Bassetti T. Corazzini L. Pavesi F. (2021a). Narratives on COVID-19 Origins and Voter Polarization. Venice: Mimeo.

[B3] Antinyan A. Bassetti T. Corazzini L. Pavesi F. (2021b). Narratives on COVID-19 and Policy Opinions: A Survey Experiment. Working Paper No. 04/WP/2021, ISSN 1827-3580. Venice: Ca' Foscari University of Venice, Department of Economics. 10.2139/ssrn.3764436

[B4] Bail C. A. Argyle L. P. Brown T. W. Bumpus J. P. Chen H. Hunzaker M. F.. (2018). Exposure to opposing views on social media can increase political polarization. Proc. Natl. Acad. Sci. U. S. A. 115, 9216–9221. 10.1073/pnas.1804840115 30154168 PMC6140520

[B5] Baliga S. Hanany E. Klibanoff P. (2013). Polarization and ambiguity. Am. Econ. Rev. 103, 3071–3083. 10.1257/aer.103.7.3071

[B6] Bartels L. M. (2002). Beyond the running tally: partisan bias in political perceptions. Polit. Behav. 24, 117–150. 10.1023/A:1021226224601

[B7] Box-Steffensmeier J. M. De Boef S. Lin T.-M. (2004). The dynamics of the Partisan Gender Gap. Am. Polit. Sci. Rev. 98, 515–528. 10.1017/S0003055404001315

[B8] Bullock J. Gerber A. Hill S. Huber G. (2015). Partisan bias in factual beliefs about politics. Quart. J. Polit. Sci. 10, 519–578. 10.1561/100.00014074

[B9] Bullock J. Lenz G. (2019). Partisan bias in surveys. Ann. Rev. Polit. Sci. 22, 325–342. 10.1146/annurev-polisci-051117-050904

[B10] Clark A. K. (2017). Updating the gender gap(s): a multilevel approach to what underpins changing cultural attitudes. Polit. Gender 13, 26–56. 10.1017/S1743923X16000520

[B11] Cohen J. (1988). Statistical Power Analysis for the Behavioral Sciences. New York, NY: Routledge.

[B12] Conover P. J. (1988). Feminists and the gender gap. J. Polit. 50, 985–1010. 10.2307/2131388

[B13] Coppock A. (2019). Generalizing from survey experiments conducted on Mechanical Turk: a replication approach. Polit. Sci. Res. Methods 7, 613–628. 10.1017/psrm.2018.10

[B14] Crow D. Jones M. (2018). Narratives as tools for influencing policy change. Policy Polit. 46, 217–234. 10.1332/030557318X15230061022899

[B15] Dimock M. Kiley J. Keeter S. Doherty C. (2014). Political Polarization in the American Public: How Increasing Ideological Uniformity and Partisan Antipathy Affect Politics, Compromise, and Everyday Life. Washington, DC: Pew Research Center.

[B16] Edlund L. Pande R. (2002). Why have women become left-wing? The political gender gap and the decline in marriage. Quart. J. Econ. 117, 917–961. 10.1162/003355302760193922

[B17] Eliaz K. Spiegler R. (2020). A model of competing narratives. Am. Econ. Rev. 110, 3786–3816. 10.1257/aer.20191099

[B18] Flynn D. J. Nyhan B. Reifler J. (2017). The nature and origins of misperceptions: understanding false and unsupported beliefs about politics. Polit. Psychol. 38, 127–150. 10.1111/pops.12394

[B19] Fryer R. G. Jr. Harms P. Jackson M. O. (2019). Updating beliefs when evidence is open to interpretation: implications for bias and polarization. J. Eur. Econ. Assoc. 17, 1470–1501 10.1093/jeea/jvy025

[B20] Gaines B. J. Kuklinski J. H. Quirk P. J. Peyton B. Verkuilen J. (2007). Same facts, different interpretations: partisan motivation and opinion on Iraq. J. Polit. 69, 957–974. 10.1111/j.1468-2508.2007.00601.x

[B21] Gentzkow M. Shapiro J. M. Taddy M. (2019). Measuring group differences in high-dimensional choices: method and application to congressional speech. Econometrica 87, 1307–1340. 10.3982/ECTA16566

[B22] Gilens M. (2001). Political ignorance and collective policy preferences. Am. Polit. Sci. Rev. 95, 379–396. 10.1017/S0003055401002222

[B23] Gillion D. Q. Ladd J. M. Meredith M. (2020). Party polarization, ideological sorting and the emergence of the US Partisan Gender Gap. Br. J. Polit. Sci. 50, 1217–1243. 10.1017/S0007123418000285

[B24] Howell W. G. West M. R. (2009). Educating the public: how information affects Americans' support for school spending and charter schools. Education Next 9, 40–48.

[B25] Huddy L. Cassese E. Lizotte M.-K. (2008). “Gender, public opinion, and political reasoning,” in Political Women and American Democracy, eds Wolbrecht C. Beckwith K. Baldez L. (New York, NY: Cambridge University Press), 31–49. 10.1017/CBO9780511790621.005

[B26] Iversen T. Rosenbluth F. (2006). The political economy of gender: explaining cross-national variation in the gender division of labor and the gender voting gap. Am. J. Polit. Sci. 50, 1–19. 10.1111/j.1540-5907.2006.00166.x

[B27] Jones J. (2019). Democratic States Exceed Republican States by Four in 2018. Gallup, 22 February. Available online at: https://news.gallup.com/poll/247025/democratic-states-exceed-republican-states-four-2018.aspx (accessed May 9, 2020).

[B28] Karadja M. Mollerstrom J. Seim D. (2017). Richer (and holier) than thou? The effect of relative income improvements on demand for redistribution. Rev. Econ. Statist. 99, 201–212. 10.1162/REST_a_00623

[B29] Kaufmann K. M. (2002). Culture wars, secular realignment, and the gender gap in party identification. Polit. Behav. 24, 283–307. 10.1023/A:1021824624892

[B30] Kaufmann K. M. Petrocik J. R. (1999). The changing politics of American men: understanding the sources of the gender gap. Am. J. Polit. Sci. 43, 864–887. 10.2307/2991838

[B31] Klar S. (2014). Partisanship in a social setting. Am. J. Polit. Sci. 58, 687–704. 10.1111/ajps.12087

[B32] Kraft P. W. Lodge M. Taber C. S. (2015). Why people “don't trust the evidence” motivated reasoning and scientific beliefs. ANNALS Am. Acad. Polit. Soc. Sci. 658, 121–133. 10.1177/0002716214554758

[B33] Kunda Z. (1990). The case for motivated reasoning. Psychol. Bullet. 108, 480–498. 10.1037/0033-2909.108.3.480 2270237

[B34] Linden A. Samuels S. J. (2013). Using balance statistics to determine the optimal number of controls in matching studies. J. Eval. Clin. Practice 19, 968–975. 10.1111/jep.12072 23910956

[B35] Loh I. Phelan G. (2019). Dimensionality and disagreement: asymptotic belief divergence in response to common information. Int. Econ. Rev. 60, 1861–1876. 10.1111/iere.12406

[B36] Lord C. G. Ross L. Lepper M. R. (1979). Biased assimilation and attitude polarization: the effects of prior theories on subsequently considered evidence. J. Personal. Soc. Psychol. 37, 2098–2109. 10.1037/0022-3514.37.11.2098

[B37] MacKinnon D. P. Fairchild A. J. Fritz M. S. (2007). Mediation analysis. Ann. Rev. Psychol. 58, 593–614. 10.1146/annurev.psych.58.110405.085542 16968208 PMC2819368

[B38] Martin G. J. Yurukoglu A. (2017). Bias in cable news: persuasion and polarization. Am. Econ. Rev. 107, 2565–2599. 10.1257/aer.20160812

[B39] Miller J. M. Saunders K. L. Farhart C. E. (2016). Conspiracy endorsement as motivated reasoning: the moderating roles of political knowledge and trust. Am. J. Polit. Sci. 60, 824–844. 10.1111/ajps.12234

[B40] Miller P. R. Conover P. J. (2015). Red and blue states of mind: Partisan hostility and voting in the United States. Polit. Res. Quart. 68, 225–239. 10.1177/1065912915577208

[B41] Morson G. S. Schapiro M. (2017). Cents and Sensibility: What Economics Can Learn from the Humanities. Princeton, NJ: Princeton University Press. 10.1515/9781400884841

[B42] Normand S. L. T. Landrum M. B. Guadagnoli E. Ayanian J. Z. Ryan T. J. Cleary P. D.. (2001). Validating recommendations for coronary angiography following an acute myocardial infarction in the elderly: a matched analysis using propensity scores. J. Clin. Epidemiol. 54, 387–398. 10.1016/S0895-4356(00)00321-8 11297888

[B43] Palan S. Schitter C. (2018). Prolific. ac—A subject pool for online experiments. J. Behav. Exp. Fin. 17, 22–27. 10.1016/j.jbef.2017.12.004

[B44] Peterson E. Iyengar S. (2020). Partisan gaps in political information and information-seeking behavior: motivated reasoning or cheerleading? Am. J. Polit. Sci. 2020:pmn7h. 10.31235/osf.io/pmn7h

[B45] Prior M. Sood G. Khanna K. (2015). You cannot be serious: the impact of accuracy incentives on partisan bias in reports of economic perceptions. Quart. J. Polit. Sci. 10, 489–518. 10.1561/100.00014127

[B46] Rubin D. B. (2001). Using propensity scores to help design observational studies: application to the tobacco litigation. Health Services Outcomes Res. Methodol. 2, 169–188. 10.1023/A:1020363010465

[B47] Schaeffer K. (2020). Nearly Three-in-Ten Americans Believe COVID-19 Was Made in a Lab. Pew Research Centre. Available online at: https://www.pewresearch.org/fact-tank/2020/04/08/nearly-three-in-ten-americans-believe-covid-19-was-made-in-a-lab (accessed May 13, 2020).

[B48] Schaffner B. F. Luks S. (2018). Misinformation or expressive responding? What an inauguration crowd can tell us about the source of political misinformation in surveys. Publ. Opin. Quart. 82, 135–147. 10.1093/poq/nfx042

[B49] Shapiro R. Y. Mahajan H. (1986). Gender differences in policy preferences: a summary of trends from the 1960s to the 1980s. Publ. Opin. Quart. 50, 42–61. 10.1086/268958

[B50] Shiller R. J. (2017). Narrative economics. Am. Econ. Rev. 107, 967–1004. 10.1257/aer.107.4.967

[B51] Shiller R. J. (2019). Narrative Economics: How Stories Go Viral and Drive Major Economic Events. Princeton, CA: Princeton University Press. 10.1515/9780691189970

[B52] Taber C. S. Lodge M. (2006). Motivated skepticism in the evaluation of political beliefs. Am. J. Polit. Sci. 50, 755–769. 10.1111/j.1540-5907.2006.00214.x

[B53] USA Facts (2020). US Coronavirus Cases and Deaths. Track COVID-19 Data Daily by State and County. Available online at: https://usafacts.org/visualizations/coronavirus-covid-19-spread-map (accessed May 15, 2020).

