# Supplementary material for: Political Narratives and the US Partisan Gender Gap
Source: Front Psychol. 2021 Jun 18;12:675684. doi: 10.3389/fpsyg.2021.675684 (PMC8253555; doi:10.3389/fpsyg.2021.675684)
Supplement: Supplementary file 1 [file Data_Sheet_1.docx]

## **Political Narratives and the US Partisan Gender Gap**

## **Supplementary Information**

Armenak Antinyan,^[[1]](#footnote-1)^† Thomas Bassetti,^[[2]](#footnote-2)^** Luca Corazzini,^[[3]](#footnote-3)^‡ Filippo Pavesi^[[4]](#footnote-4)^§

1. **Balance and Manipulation Tests**

Table A1 reports two distributional test statistics for each covariate. The first statistic assesses the balance between pairs of treatment groups in the means using the standardized difference, whereas the second statistic uses the variance ratio to check the balance in the variances. Although there is no empirical evidence suggesting a particular cut-point for these statistics, we may say that standardized differences (variance ratios) should be as close to zero (one) as possible (Linden and Samuels, 2013). In this respect, our balance test reveals that our randomization process has been successful. Indeed, the standardized differences are always less than 0.1 (i.e., the threshold value indicated in Normand et al., 2001), and the variance ratio is always between 2.0 and 0.5 as recommended by Rubin (2001).^[[5]](#footnote-5)^

| Table A1. Covariate Balance Test | | | | | | |
| --- | --- | --- | --- | --- | --- | --- |
|  | *Lab narrative vs Baseline* | | *Nature narrative vs Baseline* | | *Lab vs Nature narrative* | |
|  | Std. Diff | Var. Ratio | Std. Diff | Var. Ratio | Std. Diff | Var. Ratio |
|  | (1) | (2) | (3) | (4) | (5) | (6) |
| *male* | 0.05 | 1.00 | 0.00 | 1.00 | -0.05 | 1.00 |
| *age* | 0.01 | 0.95 | -0.05 | 0.93 | -0.06 | 0.98 |
| *income* | -0.03 | 1.19 | -0.04 | 1.20 | -0.01 | 1.01 |
| *republican state (rep)* | -0.09 | 0.95 | -0.07 | 0.96 | 0.02 | 1.01 |
| *COVID-19* | -0.05 | 0.87 | -0.02 | 0.96 | 0.03 | 1.11 |
| *lockdown* | 0.04 | 0.95 | 0.02 | 0.97 | -0.02 | 1.03 |
| *lower than high school* | 0.01 | 1.16 | -0.01 | 0.91 | -0.02 | 0.78 |
| *high school* | -0.03 | 0.98 | -0.02 | 0.99 | 0.01 | 1.01 |
| *bachelor’s degree* | -0.02 | 1.00 | 0.00 | 1.00 | 0.02 | 1.00 |
| *master's degree* | 0.06 | 1.12 | -0.01 | 0.97 | -0.07 | 0.86 |
| *doctoral degree* | 0.03 | 1.13 | 0.08 | 1.39 | 0.05 | 1.23 |
| *employed* | -0.06 | 0.76 | -0.06 | 0.78 | 0.00 | 1.02 |
| *self-employed* | 0.05 | 1.12 | 0.06 | 1.17 | 0.02 | 1.04 |
| *student* | -0.05 | 0.91 | 0.02 | 1.03 | 0.07 | 1.13 |
| *unemployed* | 0.03 | 1.05 | 0.03 | 1.06 | 0.00 | 1.01 |
| *other* | 0.01 | 1.00 | -0.05 | 1.00 | -0.07 | 1.00 |
| *metro county* | -0.04 | 1.09 | -0.03 | 1.06 | 0.01 | 0.98 |
| *republican county* | -0.03 | 0.99 | -0.05 | 0.98 | -0.02 | 0.99 |
| *marriage rate* | -0.06 | 0.67 | -0.05 | 0.65 | 0.01 | 0.97 |

Our experimental manipulation consists of exposing treated subjects to two competing narratives about COVID-19 origins. To claim any causal effect of these narratives on respondents’ political preferences, we must be sure that subjects read and correctly understood the story they were exposed to. In the experiment, we asked treated subjects to sum up in no more than two sentences the cause of COVID-19 according to the text they read. Therefore, we may carry out a quantitative content analysis to check the effectiveness of our manipulation. In particular, we use a co-occurrence network analysis to identify those words that co-occurred in treated participants’ text. This technique is widely used in textual analyses and is based on the assumption that recurrent words in a sentence are more likely to be functionally linked (Danowski, 1993; Müller and Mancuso, 2008).

Three structural measures characterize our co-occurrence networks:

1. Centrality. This measure exploits the fact that there always exists at least one shortest path between two nodes in a connected graph. Our centrality measure indicates the number of shortest paths passing through a node.
2. The minimum spanning tree (MST). The MST denotes a subset of the edges of a graph that connects all the vertices together, without any cycles and with the minimum possible total edge weight. In our representations, highlighted links correspond to stronger edges.
3. Frequency. In our graphs, larger nodes represent highly frequent words. This means that the size of the nodes is proportional to the number of times each word appeared.

Figures A1 and A2 display the co-occurrence networks for sentences written in each treatment group. Figure A1 refers to the group treated with the *Lab narrative* and indicates the use of two distinct blocks of words. The following terms form the first block: Wuhan, lab (or laboratory), bat, leak, virus, and originate. The second block involves the use of do, not, wet, market, be, cause. Figure A2 shows the co-occurrence network for words used to describe the *Nature narrative*. Here subjects focus on the fact that the virus originated from bats as a natural phenomenon. This means that respondents correctly understood the message contained in the two narratives.

**
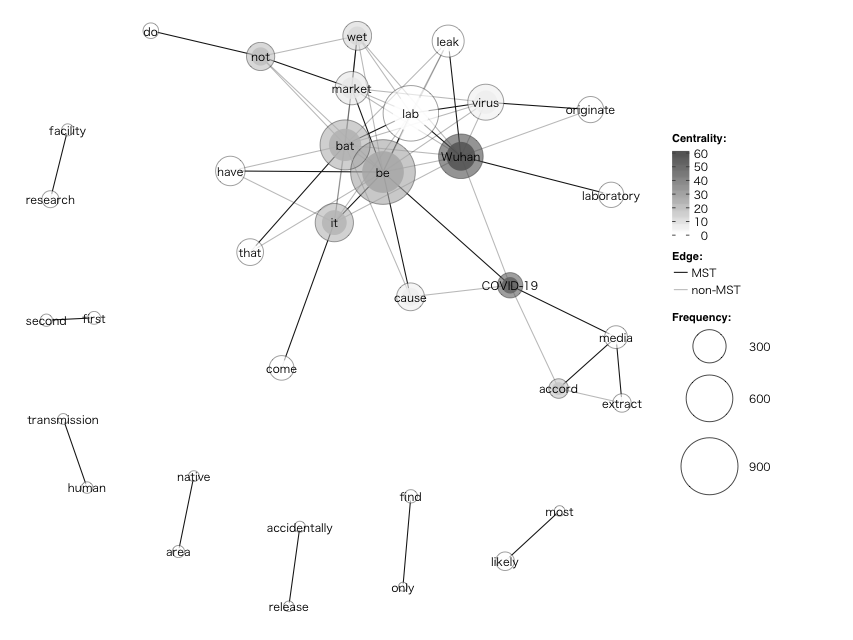
**

Figure A1. Co-occurrence Network (Lab Narrative)

*Notes:* This figure displays the co-occurrence networks for sentences that subjects exposed to the Lab narrative used to describe the text they read. Three measures of network are considered: centrality, the minimum spanning tree, and frequency.

**
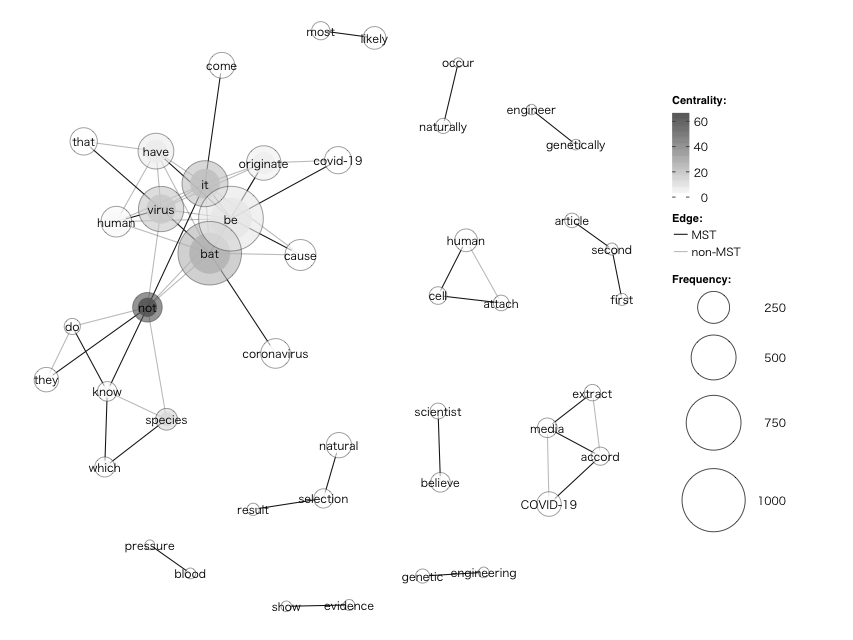
**

Figure A2. Co-occurrence Network (Nature Narrative)

*Notes*: This figure displays the co-occurrence networks for sentences that subjects exposed to the Nature narrative used to describe the text they read. Three measures of network are considered: centrality, the minimum spanning tree, and frequency.

1. **Mediation Equations**

Table B1 reports the coefficients of the mediation equations corresponding to (4). In Column 1, the dependent variable is the number of points subjects assigned to the hypothesis that COVID-19 is the consequence of a natural phenomenon in which viruses are transmitted to humans by animals. According to this column, subjects exposed to the *Lab narrative* allocate on average 8.677 point less than subjects in the Baseline group. In contrast, respondents exposed to the *Nature narrative* tend to assign on average 11.381 points more to the nature hypothesis than those in the Baseline group. By looking at the coefficient of *Nature narrative*male*, we can notice that men exposed to the *Nature narrative* are more reticent to believe in the nature hypothesis. Column 2 shows that, compared to the Baseline group, individuals treated with the *Lab narrative* assign on average 10.812 points more to the hypothesis that COVID-19 originated from an accident in a lab. Finally, Column 3 indicates that the weapon hypothesis is not sensitive to our treatments. A significant negative effect of the *Nature narrative* on the weapon hypothesis would emerge if we do not include a gender-treatment interaction term.

| Table B1. Mediation Equations | | | |
| --- | --- | --- | --- |
|  | Nature hypothesis | Accident hypothesis | Weapon hypothesis |
|  | (1) | (2) | (3) |
| *Lab narrative* | -8.684*** | 10.797*** | 0.157 |
|  | (2.487) | (1.910) | (1.075) |
| *Nature narrative* | 11.271*** | -3.400* | -1.515 |
|  | (2.473) | (1.899) | (1.069) |
| *Lab narrative*male* | -0.861 | -1.051 | -0.407 |
|  | (3.123) | (2.399) | (1.350) |
| *Nature narrative*male* | -6.577** | 1.682 | -0.409 |
|  | (3.131) | (2.404) | (1.353) |
| *male* | 5.483** | -0.108 | -1.541 |
|  | (2.198) | (1.688) | (0.950) |
| *rep* | 2.577 | -0.951 | 1.134 |
|  | (2.382) | (1.830) | (1.030) |
| *Lab narrative*rep* | -4.177 | 3.427 | -0.138 |
|  | (3.281) | (2.520) | (1.418) |
| *Nature narrative*rep* | -0.798 | -0.368 | -1.169 |
|  | (3.272) | (2.513) | (1.415) |
| *Additional controls* | Yes | Yes | Yes |
| *Observations* | 3,086 | 3,086 | 3,086 |
| *R^2^* | 0.060 | 0.066 | 0.017 |
| Notes: Coefficients of Equation (4) for potential COVID-19 causes. The other remarks about the additional controls of Table 3 apply. Standard errors are in parentheses and covariance among equations is allowed. Significance levels: *p<0.1, **p<0.05, ***p<0.01. | | | |

1. **Robustness Checks**

Table C1 contains the odds ratios of Equation (3), where the standard normal cumulative distribution function has been replaced with a logistic cumulative distribution function. These results are perfectly consistent with those reported in the main text.

Because ordered models are based on the proportional odds assumption, Table C2 shows the results of a Brant test for Column 2 of Table C1. According to this test, we only reject the proportional odds assumption for the *Lab narrative* at 10 percent of significance level. Overall, we cannot reject this assumption and therefore our estimation strategy is appropriate.

| Table C1. Political View (Ordered Logit) | | |
| --- | --- | --- |
|  | (1) | (2) |
| *Lab narrative* | 0.78** | 0.66*** |
|  | (0.09) | (0.09) |
| *Nature narrative* | 0.75** | 0.66*** |
|  | (0.08) | (0.08) |
| *Lab narrative*male* | 1.38** | 1.39** |
|  | (0.22) | (0.23) |
| *Nature narrative*male* | 1.52*** | 1.53*** |
|  | (0.24) | (0.25) |
| *male* | 1.10 | 1.09 |
|  | (0.13) | (0.12) |
| *rep* | 1.17* | 0.91 |
|  | (0.09) | (0.12) |
| *Lab narrative*rep* |  | 1.55** |
|  |  | (0.27) |
| *Nature narrative*rep* |  | 1.38* |
|  |  | (0.23) |
| *Additional controls* | Yes | Yes |
| *Observations* | 3,086 | 3,086 |
| *Pseudo-R^2^* | 0.02 | 0.02 |
| *Log-likelihood* | -4293.42 | -4289.78 |
| Notes: Ordered logit estimates of Equation (3). The other remarks about the additional controls of Table 3 apply. Robust standard errors are in parentheses. Significance levels: *p<0.1, **p<0.05, ***p<0.01. | | |

| Table C2. Brant Test of Parallel Regression Assumption | | | |
| --- | --- | --- | --- |
| Variable | $\chi^{2}$ | P-value | DF |
| *All* | 31.30 | 0.145 | 24 |
| *Lab narrative* | 7.12 | 0.068 | 3 |
| *Nature narrative* | 3.63 | 0.304 | 3 |
| *Lab narrative*male* | 5.1 | 0.165 | 3 |
| *Nature narrative*male* | 0.76 | 0.86 | 3 |
| *male* | 4.98 | 0.173 | 3 |
| *rep* | 2.15 | 0.543 | 3 |
| *Lab narrative*rep* | 3.03 | 0.387 | 3 |
| *Nature narrative*rep* | 1.23 | 0.747 | 3 |

In Table C3, we considered switching states as a separate class. More specifically, we separated those states that in the last election kept the political orientation indicated by the 2018 Gallup report from those that changed their blue/red status. Nonetheless, our results continue to hold.

| Table C3. Political Views and Switching States (OLS and Ordered Probit) | | | | |
| --- | --- | --- | --- | --- |
|  | OLS | | Ordered Probit | |
|  | (1) | (2) | (3) | (4) |
| *Lab narrative* | -0.158** | -0.253*** | -0.174*** | -0.270*** |
|  | (0.063) | (0.073) | (0.067) | (0.078) |
| *Nature narrative* | -0.167*** | -0.243*** | -0.178*** | -0.257*** |
|  | (0.062) | (0.072) | (0.066) | (0.076) |
| *Lab narrative*male* | 0.192** | 0.195** | 0.211** | 0.214** |
|  | (0.089) | (0.089) | (0.093) | (0.093) |
| *Nature narrative*male* | 0.240*** | 0.243*** | 0.257*** | 0.260*** |
|  | (0.089) | (0.089) | (0.093) | (0.093) |
| *male* | 0.038 | 0.035 | 0.037 | 0.035 |
|  | (0.063) | (0.063) | (0.065) | (0.065) |
| *rep* | 0.129*** | -0.013 | 0.128*** | -0.017 |
|  | (0.047) | (0.074) | (0.049) | (0.077) |
| *switching* | 0.254*** | 0.171 | 0.257*** | 0.171 |
|  | (0.063) | (0.107) | (0.065) | (0.109) |
| *Lab narrative*rep* |  | 0.261** |  | 0.262** |
|  |  | (0.104) |  | (0.108) |
| *Lab narrative*switching* | | 0.104 | 0.103 |  |
|  |  | (0.144) |  | (0.148) |
| *Nature narrative*rep* | | 0.176* | 0.176* |  |
|  |  | (0.100) |  | (0.104) |
| *Nature narrative*switching* | | 0.149 | 0.149 |  |
|  |  | (0.151) |  | (0.155) |
| *Constant* | 2.114*** | 2.193*** |  |  |
|  | (0.182) | (0.185) |  |  |
| *Additional controls* | Yes | Yes | Yes | Yes |
| *Observations* | 3,086 | 3,086 | 3,086 | 3,086 |
| *R^2^ and Pseudo-R^2^* | 0.065 | 0.067 | 0.023 | 0.023 |
| *Log-likelihood* |  |  | -4283.039 | -4279.507 |
| Notes: Estimates of Equations (1) and (3), considering switching states separately. The other remarks about the additional controls of Table 3 apply. Robust standard errors are in parentheses. Significance levels: *p<0.1, **p<0.05, ***p<0.01. | | | | |
|  |  |  |  |  |

In Table C4, we re-estimated Equations (1) and (3) substituting COVID-19 incidence rates and self-reported lockdown restrictions with COVID-19 mortality rates and official stay-at-home rules. In this respect, we re-classified states using Figure 1 from the CDC Morbidity and Mortality Weekly Report (Moreland et al., 2020). Our results continue to hold even when we consider four different categories of stay-at-home restrictions: 1=no restriction; 2=advisory order; 3=mandatory for persons at risk; 4=mandatory for all).

| Table C4. Political View (OLS and Ordered Probit) | | | | |
| --- | --- | --- | --- | --- |
|  | OLS | | Ordered Probit | |
|  | (1) | (2) | (3) | (4) |
| *Lab narrative* | -0.159** | -0.245*** | -0.175*** | -0.262*** |
|  | (0.064) | (0.071) | (0.067) | (0.075) |
| *Nature narrative* | -0.172*** | -0.234*** | -0.182*** | -0.247*** |
|  | (0.062) | (0.070) | (0.066) | (0.074) |
| *Lab narrative***male* | 0.190** | 0.192** | 0.207** | 0.211** |
|  | (0.090) | (0.090) | (0.093) | (0.093) |
| *Nature narrative***male* | 0.239*** | 0.243*** | 0.255*** | 0.258*** |
|  | (0.089) | (0.089) | (0.093) | (0.093) |
| *male* | 0.040 | 0.038 | 0.039 | 0.037 |
|  | (0.063) | (0.063) | (0.065) | (0.065) |
| *rep* | 0.098** | -0.035 | 0.098** | -0.037 |
|  | (0.046) | (0.071) | (0.048) | (0.073) |
| *Lab narrative***rep* |  | 0.240** |  | 0.241** |
|  |  | (0.097) |  | (0.100) |
| *Nature narrative***rep* |  | 0.168* |  | 0.174* |
|  |  | (0.095) |  | (0.098) |
| *Constant* | 1.959*** | 2.035*** |  |  |
|  | (0.225) | (0.227) |  |  |
| *Additional controls* | Yes | Yes | Yes | Yes |
| *Observations* | 3,086 | 3,086 | 3,086 | 3,086 |
| *R^2^ and Pseudo-R^2^* | 0.061 | 0.063 | 0.021 | 0.022 |
| *Log-likelihood* |  |  | -4289.444 | -4286.162 |
| Notes: Estimates of Equations (1) and (3), considering as control variables COVID-19 death rates instead of incidence rates and official stay-at-home rules instead of self-reported lockdown restrictions. The other remarks about the additional controls of Table 3 apply. Robust standard errors in parentheses. Significance levels: *p<0.1, **p<0.05, ***p<0.01. | | | | |

1. **Narratives**

Below we provide the full text of each narrative, each of which is based on coverage from two media sources on the opposite side of the US political spectrum (CNN and Fox News). Each extract has the feature of being backed by research, implying that the only salient difference between the extracts is the narrative itself and not its formal validation. Moreover, we chose to hide the name of the specific research cited in the articles from which the extracts were taken to control for potential reputation effects. Three of the extracts were taken directly from the websites of the two media outlets, while one was taken from the Daily Caller, a source that is however directly connected to Fox News. The reasons for this choice were the following: 1) the Daily Caller was directly owned by the Fox News anchorman Tucker Carlson that the article is quoting at the time the experiment was run; 2) the Daily Caller article directly quotes Fox News and actually embeds the Fox News video that includes Carlson’s speech that is cited in the article; 3) Tucker Carlson’s speech, like the other extracts, contains a narrative that is research based and therefore in no way less formally validated than the other extracts. The reason we chose this particular extract is that at the time the experiment was run, the other Fox News stories making the same argument were more based on political statements rather than on research, and therefore less homogeneous on this dimension in comparison to the other extracts.

**D.1. The Lab Narrative**

*Media extract 1*^^[[6]](#footnote-6)^^

A potentially explosive theory — first posed by two Chinese researchers in early February holds that the origin of COVID-19 traces back to an accident in one of two labs near the Wuhan market that work with bats. Most of the experts interviewed for this story discounted the theory — whose progenitors reportedly withdrew their paper — saying it wasn't supported by evidence. The theory has also been strenuously denied by the Chinese government and one of the labs. But one expert, a chemical biology professor and bio-weapons expert at an important US University, has suggested to several media outlets that the lab-accident theory has credence. "The possibility that the virus entered humans through a laboratory accident cannot and should not be dismissed," said the doctor in an email.

*Media extract 2^^[[7]](#footnote-7)^^*

Despite previous reports that coronavirus had been traced to bats, most likely the kind that could be found in wet markets like the one in Wuhan, a new report from an important Chinese University suggests that there’s a more likely scenario — a leak from a lab. The report detailed the tracing of COVID-19 to the intermediate horseshoe bat — a bat that they confirmed was not available at the Wuhan wet market and did not live locally. In fact, the report noted that native populations were no closer than 600 miles away from the first known cases, making a natural transmission from bat to human appear more unlikely.

The only place those particular bats existed locally was inside a research facility — which was just several hundred yards from the Wuhan wet market — and the paper’s ultimate conclusion was that the coronavirus pandemic had likely been the result of a leak from the lab: “The killer coronavirus probably originated from a laboratory in Wuhan.”

**D.2. The Nature Narrative**

*Media extract 3*^^[[8]](#footnote-8)^^

In early February, Chinese researchers published an article in a top science journal that concluded the “COVID-19 is 96% identical at the whole-genome level to a bat coronavirus." Later that month, 27 public health scientists from across the United States and the world wrote a letter in another leading scientific journal. In the piece, the experts cited scientific evidence that support the theory that "overwhelmingly conclude that this coronavirus originated in wildlife, as have so many other emerging pathogens. "We're very confident that the origin of Covid-19 is in bats," the president of a health nonprofit that tracks zoonotic spillover. "We just don't know where exactly it originated – which bat species exactly. And we don't know how many others there are out there that could emerge in the future.“

*Media extract 4^[[9]](#footnote-9)^*

A group of researchers compared the genome of this novel coronavirus with the seven other coronaviruses known to infect humans: SARS, MERS and COVID-19, which can cause severe disease. That analysis showed that the "hook" part of the spike had evolved to target a receptor on the outside of human cells called ACE2, which is involved in blood pressure regulation. It is so effective at attaching to human cells that the researchers said the spike proteins were the result of natural selection and not genetic engineering. The overall molecular structure of this virus is distinct from the known coronaviruses and instead most closely resembles viruses found in bats and pangolins that had been little studied and never known to cause humans any harm.

1. **Questionnaire**

*[The questionnaire was written in American English. The first block of Lab narrative included the two extracts reported in A.4.1. The first block of Nature narrative included the two extracts reported in A.4.2. The first block was not included in the No story. The order of the questions on policy domains in block 2 was randomized across subjects.]*

*[Screen 1: Welcome screen]*

Welcome to this survey. We are conducting a short academic study about COVID-19 and other issues. The questionnaire is anonymous, and the answers will be analyzed in aggregate form for research purposes only. Please answer all questions accurately. There are no correct answers! Therefore, we ask you to answer sincerely on the basis of your own opinions. The survey will take around 10 minutes. Thank you for your help. Please do not hesitate to contact us for further information on our study:

Armenak Antinyan (antinyan.armenak@gmail.com);

Thomas Bassetti (thomas.bassetti@unipd.it);

Luca Corazzini (luca.corazzini@unive.it);

Filippo Pavesi (fpavesi@liuc.it)

Please choose, whether you would like to continue with the study or quit. Please note that if you decide to quit you will not be paid. If you do not give your consent, please do not forget to return your submission on Prolific by selecting the ’Stop without completing’ button.

[] I GIVE MY CONSENT, and I want to continue with the study.

[] I DO NOT GIVE MY CONSENT, and I want to quit the study.

*[Screen 2: Prolific identification]*

Provide your Prolific identification number.

*[Screen 3 (Block 1): Introduction to the media extracts]*

In the next page, you will find extracts from articles in the US media about COVID-19. Please read them carefully before answering the questions that follow.

Disclaimer: Views, thoughts, and opinions expressed in the following paragraphs do not necessarily reflect the view of the researchers involved in the research project.

*[Screen 4 (Block 1): Media extracts]*

*[Screen 5 (Block 1): Manipulation check]*

Please summarize in no more than two sentences what has caused COVID-19 according to the media extracts in the previous screen.

*[Screen 6 (Block 2): Questions on policy domains]*

Foreign trade represents more of an opportunity than a threat for the US economy in the post COVID-19 recovery. On a scale from 1 (Completely agree) to 5 (Completely disagree) indicate how much you agree with the previous statement.

[] 1 Completely agree

[] 2 Agree

[] 3 Neither agree, nor disagree

[] 4 Disagree

[] 5 Completely disagree

*[Screen 7 (Block 2)]*

Science is improving our lives, and, in the post COVID-19 recovery, scientific progress should rapidly continue despite potential ethical and safety concerns. On a scale from 1 (Completely agree) to 5 (Completely disagree) indicate how much you agree with the previous statement.

[] 1 Completely agree

[] 2 Agree

[] 3 Neither agree, nor disagree

[] 4 Disagree

[] 5 Completely disagree

*[Screen 8 (Block 2)]*

Preventing climate change should be given priority in the post COVID-19 recovery, even if it causes slower economic growth and some loss of jobs. On a scale from 1 (Completely agree) to 5 (Completely disagree) indicate how much you agree with the previous statement.

[] 1 Completely agree

[] 2 Agree

[] 3 Neither agree, nor disagree

[] 4 Disagree

[] 5 Completely disagree

*[Screen 9 (Block 3): Questions on what caused COVID-19]*

We now want to investigate your opinions about the causes of COVID-19. Please allocate 100 points across potential causes of COVID-19. The higher the points allocated to a given cause, the more you believe that specific cause provoked COVID-19.

[] The virus originated from an accident in a Lab:

[] The virus originated in nature as a result of natural processes:

[] The virus is a weapon the countries use against each other:

[] Other reasons:

Total:

*[Screen 10 (Block 3)]*

According to your response to the previous question, you believe that COVID-19 was caused by "other reasons". Please detail your answer below.

[Screen 11 (Block 4): Socio-economic and demographic questions]

In the next future, how willing would you be to get vaccinated against viruses other than COVID-19 (e.g., influenza and other infectious viruses) on a scale from 1 (Extremely willing) to 5 (Extremely unwilling)?

[] 1 Extremely willing

[] 2 Willing

[] 3 Neither willing, nor unwilling

[] 4 Unwilling

[] 5 Extremely unwilling

*[Screen 12 (Block 4)]*

What is your gender?

[] Male

[] Female

[] Other

[Screen 13 (Block 4)]

What is your age?

*[Screen 14 (Block 4)]*

Which of the following describes your situation the best? Please think about the activity or situation which is primary for you.

[] Unemployed

[] Student

[] Working either part-time or full-time

[] Self-employed

[] Other

*[Screen 15 (Block 4)]*

What is the highest level of education you achieved to date?

[] No formal education

[] Lower than a high school diploma

[] High school diploma

[] Bachelor degree

[] Master’s degree

[] Doctoral degree

*[Screen 16 (Block 4)]*

Imagine an income scale on which 1 indicates the lowest income group and 10 the highest income group in the United States. We would like to know in what group your household is. Please, specify the appropriate number, counting all wages, salaries, pensions and other incomes earned.

[] 1 Lowest income group

[] 2

[] 3

[] 4

[] 5

[] 6

[] 7

[] 8

[] 9

[] 10 Highest income group

*[Screen 17 (Block 4)]*

Are lockdown restrictions currently in place where you are living?

[] Yes

[] No

*[Screen 18 (Block 4)]*

How would you describe your political views?

[] Very conservative

[] Conservative

[] Moderate

[] Liberal

[] Very liberal

*[Screen 19 (Block 4)]*

In which US state are you residing in?

*[Screen 20 (Block 4)]*

On a typical day, about how much time do you spend watching, reading or listening to news about politics and current affairs? Please give your answer in minutes.

1. **Main Results with Controls’ Coefficients**

Table F.1 reports the estimates presented in Table 3 of the manuscript together with the coefficients of control variables. According to this table, the statistically significant controls are income, education, age and county’s political orientation.

| Table F.1. Political View (OLS and Ordered Probit) | | | | |
| --- | --- | --- | --- | --- |
|  | OLS | | Ordered Probit | |
|  | (1) | (2) | (3) | (4) |
| *Lab narrative* | -0.154** | -0.241*** | -0.169** | -0.257*** |
|  | (0.064) | (0.071) | (0.067) | (0.075) |
| *Nature narrative* | -0.165*** | -0.229*** | -0.176*** | -0.242*** |
|  | (0.062) | (0.070) | (0.066) | (0.074) |
| *Lab narrative***male* | 0.183** | 0.186** | 0.201** | 0.204** |
|  | (0.090) | (0.090) | (0.093) | (0.093) |
| *Nature narrative***male* | 0.232*** | 0.235*** | 0.248*** | 0.251*** |
|  | (0.089) | (0.089) | (0.093) | (0.093) |
| *male* | 0.045 | 0.042 | 0.044 | 0.041 |
|  | (0.063) | (0.063) | (0.065) | (0.065) |
| *rep* | 0.092** | -0.042 | 0.091** | -0.046 |
|  | (0.045) | (0.070) | (0.046) | (0.072) |
| *Lab narrative***rep* |  | 0.242** |  | 0.243** |
|  |  | (0.097) |  | (0.100) |
| *Nature narrative***rep* |  | 0.171* |  | 0.177* |
|  |  | (0.095) |  | (0.098) |
| *Education* | -0.108*** | -0.108*** | -0.114*** | -0.114*** |
|  | (0.025) | (0.025) | (0.026) | (0.026) |
| *Lockdown* | -0.072 | -0.070 | -0.076 | -0.074 |
|  | (0.045) | (0.045) | (0.047) | (0.047) |
| *Income* | 0.043*** | 0.042*** | 0.043*** | 0.042*** |
|  | (0.011) | (0.011) | (0.012) | (0.012) |
| *Age* | 0.012*** | 0.012*** | 0.012*** | 0.012*** |
|  | (0.002) | (0.002) | (0.002) | (0.002) |
| *Self-employed* | -0.026 | -0.030 | -0.033 | -0.038 |
|  | (0.114) | (0.114) | (0.116) | (0.117) |
| *Student* | -0.069 | -0.075 | -0.070 | -0.076 |
|  | (0.117) | (0.117) | (0.120) | (0.120) |
| *Unemployed* | -0.124 | -0.132 | -0.137 | -0.145 |
|  | (0.110) | (0.110) | (0.113) | (0.113) |
| *Other* | 0.039 | 0.036 | 0.039 | 0.035 |
|  | (0.104) | (0.105) | (0.106) | (0.107) |
| *COVID-19* | -0.213 | -0.224 | -0.228 | -0.239 |
|  | (0.155) | (0.154) | (0.164) | (0.164) |
| *Urban county* | -0.012 | -0.016 | -0.003 | -0.008 |
|  | (0.059) | (0.058) | (0.061) | (0.061) |
| *Republican county* | 0.170*** | 0.169*** | 0.171*** | 0.170*** |
|  | (0.043) | (0.043) | (0.044) | (0.044) |
| *Marriage rate* | 0.003 | 0.002 | 0.002 | 0.002 |
|  | (0.009) | (0.009) | (0.009) | (0.009) |
| *Constant* | 2.124*** | 2.196*** |  |  |
|  | (0.183) | (0.185) |  |  |
| *Observations* | 3,086 | 3,086 | 3,086 | 3,086 |
| *R^2^ and Pseudo-R^2^* | 0.061 | 0.063 | 0.021 | 0.022 |
| *Log-likelihood* |  |  | -4290.107 | -4286.770 |
| Notes: Coefficients of Equations (1) and (3). Robust standard errors in parentheses. Significance levels: *p<0.1, **p<0.05, ***p<0.01. | | | | |

**References**

Danowski, J. 1993. Network analysis of message content. In: Richards, D., Barnett, G. (Eds.). Progress in Communication Science. Norwood, N.J.: Ablex, 197-221

Linden, A., and Samuels, S. J. (2013). Using balance statistics to determine the optimal number of controls in matching studies. J. Eval. Clin. Practice 19, 968–975. doi: 10.1111/jep.12072

Moreland, A., Herlihy, C., Tynan, M.A., et al. 2020. Timing of State and Territorial COVID-19 Stay-at-Home Orders and Changes in Population Movement — United States, March 1–May 31, 2020. MMWR Morb Mortal Wkly Rep 69: 1198–1203. doi: http://dx.doi.org/10.15585/mmwr.mm6935a2

Müller, H., Mancuso, F. 2008. Identification and analysis of co-occurrence networks with NetCutter. PLoS One 3(9): p.e3178. doi: 10.1371/journal.pone.0003178

Normand, S.L.T., Landrum, M.B., Guadagnoli, E., Ayanian, J.Z., Ryan, T.J., Cleary, P.D., McNeil, B.J. 2001. Validating recommendations for coronary angiography following an acute myocardial infarction in the elderly: a matched analysis using propensity scores. Journal of Clinical Epidemiology 54: 387-398. doi: 10.1016/S0895-4356(00)00321-8

Rubin, D.B. 2001. Using propensity scores to help design observational studies: application to the tobacco litigation. Health Services & Outcomes Research Methodology 2: 169-188.

1. †Wenlan School of Business, Zhongnan University of Economics and Law, Nanhu Avenue 182, Wuhan 430073, P.R. China and Cardiff Business School, Cardiff University, Aberconway Building, Colum Drive, Cardiff CF10 3EU, Wales. E-mail: [antinyan.armenak@gmail.com](mailto:antinyan.armenak@gmail.com), Tel: *+86 027 88387186, Fax: +86 027 88387186*. [↑](#footnote-ref-1)
2. ^**^ University of Padua, Department of Economics and Management “Marco Fanno”, Via del Santo 33, 35123, Padua, Italy. Email: [thomas.bassetti@unipd.it](mailto:thomas.bassetti@unipd.it). [↑](#footnote-ref-2)
3. ‡*Corresponding author*. Department of Economics and VERA (Venice centre in Economic and Risk Analytics for public policies), University of Venice “Ca’ Foscari,” Cannaregio, 821, 30121 Venezia (VE), Italy. Email: [luca.corazzini@unive.it](mailto:luca.corazzini@unive.it). [↑](#footnote-ref-3)
4. §School of Economics and Management, University “Carlo Cattaneo” - LIUC, C.so Matteotti, 22, 21053 Castellanza (VA), Italy, and Stevens Institute of Technology, School of Business, Hoboken, NJ, USA. E-mail: [fpavesi@liuc.it](mailto:fpavesi@liuc.it). [↑](#footnote-ref-4)
5. Rubin (2001) indicates a threshold value for the standardized difference of 0.25, which is even greater than the one proposed by Normand et al. (2001). [↑](#footnote-ref-5)
6. CNN, April 6, 2020: https://edition.cnn.com/2020/04/06/us/coronavirus-scientists-debate-origin-theories-invs/index.html [↑](#footnote-ref-6)
7. Dailycaller, March 31, 2020, paraphrasing a speech from Tucker Carlson (Fox News): https://dailycaller.com/2020/03/31/coronavirus-lab-wuhan-tucker-carlson-report/ [↑](#footnote-ref-7)
8. CNN, April 6, 2020: https://edition.cnn.com/2020/04/06/us/coronavirus-scientists-debate-origin-theories-invs/index.html [↑](#footnote-ref-8)
9. Fox News, March 24, 2020: https://www.foxnews.com/science/the-coronavirus-did-not-escape-from-a-lab-heres-how-we-know [↑](#footnote-ref-9)
